# Machine learning based identification potential feature genes for prediction of drug efficacy in nonalcoholic steatohepatitis animal model

**DOI:** 10.1186/s12944-024-02231-9

**Published:** 2024-08-24

**Authors:** Marwa Matboli, Ibrahim Abdelbaky, Abdelrahman Khaled, Radwa Khaled, Shaimaa Hamady, Laila M. Farid, Mariam B. Abouelkhair, Noha E. El-Attar, Mohamed Farag Fathallah, Manal S. Abd EL Hamid, Gena M. Elmakromy, Marwa Ali

**Affiliations:** 1 Medical Biochemistry and Molecular Biology Department, Faculty of Medicine, Ain Shams University, Cairo, Egypt; 2 Artificial Intelligence Department, Faculty of Computers and Artificial Intelligence, Benha University, Benha City, Egypt; 3 Bioinformatics Group, Center of Informatics Sciences (CIS), School of Information Technology and Computer Sciences, Nile University, Giza, Egypt; 4 Biotechnology/Biomolecular Chemistry Department, Faculty of Science, Cairo University, Cairo, Egypt; 5 Basic Sciences Department, Modern University for Technology and Information, Cairo, Egypt; 6 Faculty of Science, Ain Shams University, Cairo, Egypt; 7 Faculty of Medicine, Ain Shams University, Cairo, Egypt; 8 Information System Department, Faculty of Computers and Artificial Intelligence, Benha University, Benha City, Egypt; 9 Faculty of Artificial Intelligence, Delta University for Science and Technology, Gamasa, 35712 Egypt; 10 Medical Pathology Department, Faculty of Medicine, Cairo University, Cairo, Egypt; 11 Medical Physiology Department, Faculty of Medicine, Ain Shams University, Cairo, Egypt; 12 Endocrinology & Diabetes Mellitus Unit, Department of Internal Medicine, Badr University in Cairo, Badr City, Egypt

**Keywords:** NASH, MAFLD, Machine learning, Drug efficacy non-coding RNA, Biomarkers

## Abstract

**Background:**

Nonalcoholic Steatohepatitis (NASH) results from complex liver conditions involving metabolic, inflammatory, and fibrogenic processes. Despite its burden, there has been a lack of any approved food-and-drug administration therapy up till now.

**Purpose:**

Utilizing machine learning (ML) algorithms, the study aims to identify reliable potential genes to accurately predict the treatment response in the NASH animal model using biochemical and molecular markers retrieved using bioinformatics techniques.

**Methods:**

The NASH-induced rat models were administered various microbiome-targeted therapies and herbal drugs for 12 weeks, these drugs resulted in reducing hepatic lipid accumulation, liver inflammation, and histopathological changes. The ML model was trained and tested based on the Histopathological NASH score (HPS); while (0–4) HPS considered Improved NASH and (5–8) considered non-improved, confirmed through rats’ liver histopathological examination, incorporates 34 features comprising 20 molecular markers (mRNAs-microRNAs-Long non-coding-RNAs) and 14 biochemical markers that are highly enriched in NASH pathogenesis. Six different ML models were used in the proposed model for the prediction of NASH improvement, with Gradient Boosting demonstrating the highest accuracy of 98% in predicting NASH drug response.

**Findings:**

Following a gradual reduction in features, the outcomes demonstrated superior performance when employing the Random Forest classifier, yielding an accuracy of 98.4%. The principal selected molecular features included *YAP1, LATS1, NF2, SRD5A3-AS1, FOXA2, TEAD2*, miR-650, MMP14, ITGB1, and miR-6881-5P, while the biochemical markers comprised triglycerides (TG), ALT, ALP, total bilirubin (T. Bilirubin), alpha-fetoprotein (AFP), and low-density lipoprotein cholesterol (LDL-C).

**Conclusion:**

This study introduced an ML model incorporating 16 noninvasive features, including molecular and biochemical signatures, which achieved high performance and accuracy in detecting NASH improvement. This model could potentially be used as diagnostic tools and to identify target therapies.

**Supplementary Information:**

The online version contains supplementary material available at 10.1186/s12944-024-02231-9.

## Introduction

Metabolic dysfunction-related hepatic condition (MDHC), previously identified as a Non-alcoholic fatty liver ailment (NAFLA), impacts approximately 25% of adults worldwide and emerged as a prevalent chronic hepatic ailment on a global scale [1, 2]. It is strongly associated with metabolic abnormalities like overweightness, cardiovascular risk, and type-2-diabetes mellitus (T2DM) [3, 4]. Its pathogenesis involves a multifaceted complex phenotypic and microscopic spectrum from basic fatty liver, inflammatory steatohepatitis, and fatty liver with necroinflammatory lesions, non-alcoholic steatohepatitis (NASH) the harbinger of the progressive fibrosis and cirrhotic liver and ultimately hepatocellular carcinoma (HCC), increasing the likelihoods of liver transplantation [5, 6]. With no specific pharmacotherapy approved yet directed to MDHC /NASH [7].

The intricate molecular mechanisms underlying the development of NASH continue to be largely elusive [8]. However, the widely embraced "multiple hit" hypothesis provides valuable insights [9]. This hypothesis suggests that NASH pathology involves a combination of various destructive events, such as liver fat buildup, insulin resistance, inflammation, impaired adipose tissue, altered gut microbiota, and genetic or epigenetic factors [10]. Inflammation is a key driver of NASH [11]. It is currently believed that innate immune responses are essential in maintaining hepatic inflammation in NASH [12]. Moreover, the incidence of hepatic fibrosis emerges as an indicator of NASH-related mortality [13].

The absence of a decisive therapy for NASH poses a significant challenge [14]. While lifestyle interventions, such as weight loss and managing comorbidities, remain the gold standard for NASH treatment in individuals with cirrhosis and obesity or overweightness, there is a pressing need for more effective solutions [15]. Various drugs have been tested or are being developed, targeting different aspects within and outside the liver, with the goal of improving steatohepatitis and fibrosis [16, 17]. The ongoing efforts in therapy development for MDHC and NASH primarily revolve around addressing metabolic imbalances, oxidative stress, and innate immunity [18].

The involvement of the gut microbiota in developing MDHC/NASH has been an active research entity [19]. shifts in gut microbiota composition, disruption of the gut barrier, and liver inflammation are key factors in the pathogenesis [20]. Thus, targeting the gut microbiota has been a potential approach for pharmacological treatments of MDHC [21]. The administration of probiotics has demonstrated promising results in reducing intra-hepatic triglyceride content and aspartate aminotransferase levels in NASH patients [22]. Furthermore, fecal microbiota transplantation has shown promising outcomes in alleviating steatohepatitis in animal models resulting in a significant reduction in the proinflammatory cytokines, intrahepatic lipid accumulation, as well as the NAS (MDHC Activity Score) [23–25].

Numerous drugs have undergone clinical trials for NASH treatment, but most studies have been limited in size and duration, making repeated biopsies impractical for trial participants [26, 27]. As a result, alternative methods for assessing drugs’ potential efficacy have become essential. Recent advancements have been made in utilizing non-invasive biomarkers in the initial phases of drug development [28] encompassing a range of metabolic parameters like lipid metabolism, insulin resistance, systemic inflammation, and hepatic lipogenesis, that are employed for confirming the therapeutic effects of NASH drugs in initial trials [29]. By integrating liver enzymes, ultrasound, and molecular biomarkers, researchers can effectively monitor treatment response in NASH patients, sparing them from the discomfort and potential complications associated with repeated invasive procedures [30].

The use of animal models in biological research and drug development is a long-standing practice due to several key reasons. Firstly, animal models offer significant physiological and anatomical similarities to humans, particularly mammals, enabling the study of complex immune responses and multi-tissue interactions that are crucial for understanding drug efficacy and safety. This similarity helps inform the design of clinical trials and mitigates the risk of adverse effects in human subjects. Rodents, in particular, share orthologous genes and physiological homologies with humans, making them especially suitable for studying human pathologies [31]. Secondly, the controlled experimental environment provided by animal models allows researchers to manipulate variables and isolate specific mechanisms, which can be challenging in studies involving humans due to genetic and environmental variability [32]. Finally, ethical and logistical constraints in human studies, especially those involving invasive procedures, underscore the practical importance of animal models for initial hypothesis testing and therapeutic evaluation prior to clinical trials [31]. Therefore, the models facilitate detailed mechanistic investigations into pathogenic processes such as NASH, including lipid metabolism and inflammatory signaling pathways, which are difficult to explore directly in human subjects.

Through extensive research on animal models, the authors have made significant strides in the search for a prophylactic drug for NASH. These investigations focused on various approaches, including targeting the liver-gut axis to modulate the gut microbiota using substances like kefir, the probiotic "Flora 20–14 Ultra Strength," the prebiotic inulin fiber "Greena," and synbiotics (the mixture of both probiotic and prebiotic). Additionally, anti-fibrotic strategies that directly addressed hepatic stellate cell inflammation, such as Mutaflor, were explored, moreover, rosavin was used to hinder inflammatory cell recruitment or block inflammatory signaling. To gain deeper insights, a panel of gene measurements involved in NASH pathogenesis and its epigenetic regulators were implemented. Alongside biochemical, histological, and immunohistochemistry analyses, these comprehensive evaluations provided valuable indications of the therapeutic candidates' biological actions and potential clinical benefits [33–36].

Machine learning (ML) allows autonomous learning from data that aided a variety of genomics studies [37–40]. Drug response prediction (DRP), a specialized application of ML, forecasts the phenotypic reactions of biological specimens using their molecular [41, 42]. These predictors often provide valuable insights [43]. Another significant research challenge is determining how a clinical drug will react, or how sensitive it will be, to a particular form of cancer [44]. Accurate prediction of clinical drug responses allows clinicians to comprehend variations in drug sensitivity outcomes among patients, ultimately reducing the time and costs involved in identifying effective drug candidates [45].

This study aimed to leverage machine learning (ML) algorithms to identify key genes that can reliably predict treatment responses in an animal model of NASH. By integrating biochemical, and molecular markers obtained through bioinformatics techniques, the research seeks to evaluate the efficacy of microbiome-targeted therapies and herbal drugs in managing NASH. The primary objective is to elucidate the genetic and epigenetic RNA networks involved in NASH progression and determine their potential as predictive markers for drug responses. Through rigorous validation of established biochemical-RNA signature networks in a larger animal cohort using bioinformatics and ML methodologies, the study aimed to enhance the accuracy of predicting drug efficacy in NASH treatment. Ultimately, this research underscores the significance of RNA-based predictors in advancing personalized treatment strategies for NASH.

## Materials and methods

### Bioinformatic‑based selection of biochemical-RNA signatures

Selection of the candidate RNAs was done by searching for RNA species (mRNA, miRNAs, and lncRNAs) significantly expressed in NASH and contribute to pathogenic mechanisms driving the disease. Firstly, the KEGG DISEASE Database^1^ was utilized to visualize the pathogenic pathways and processes involved in NASH genesis (Appendix Fig. A.1). Then, the differential expressed genes (DEGS) that are differentially expressed in NASH were retrieved from the gene chip datasets GSE164760, GSE24807, and GSE126848 (Appendix Table A.1) of the Gene Expression Omnibus (GEO) database.^2^ From the retrieved significant genes, Yes Associated Protein 1 (*YAP1*), Neurofibromin 2 (*NF2*), Large tumor suppressor 2 (*LATS2*), TEA Domain Transcription Factor 2 (*TEAD2*), Forkhead box protein A2 (*FOXA2*), Angiomotin Like 2 (*AMOTL2*), SRY-Box Transcription Factor 11 (*SOX11*), Large tumor suppressor 1 (*LATS1*), Mothers Against Decapentaplegic Homolog 4 (*SMAD4*), Matrix metalloproteinase 14 (MMP14), Heat shock protein Family D Member 1 (*HSPD1*), Integrin Subunit Beta 1 (*ITGB1*) Tumor necrosis factor (*TNF*) were chosen. These genes were selected based on validation by public databases and their involvement in pathogenic mechanisms such as Hippo signaling, transforming growth factor-β (TGF-β) signaling, TNF signaling pathway, apoptosis, oxidative stress, and inflammatory response (Appendix Fig. A.2). Moreover, all these selected mRNAs were validated for their correlation to gut microbiota using the Encyclopedia of gut microbiota^3^ (Appendix Fig. A.3).

The miRNAs and lncRNAs that modify the chosen candidate mRNAs were obtained from two databases: miRWalk^4^ and DIANA-LncBase.^5^ After evaluating their high interaction with the candidate mRNAs and their involvement in NASH-related pathogenic mechanisms, miR-1205, miR-6881-5p, miR-650, miR-6807-5p, as well as LncRNAs-RPARP-AS1, SPARCL1-1:2, and SRD5A3-AS1 were selected (Appendix Figs. A.4-A.6).

Selection of the biochemical signatures was done by searching target effector proteins interacting with the selected candidate genes and strongly contributed to the inflammatory and fibrotic processes of NASH progression. As a result, TGF-β1 and interleukin-6 (IL6) were obtained (Appendix Fig. A.2). The robustness of the correlation between RNAs and biochemical signatures was evaluated using the STRING database, which indicated a significant enrichment in protein–protein interactions (PPI) with a highly noteworthy *P*-value of 3.33e-15.

### Drugs and chemicals

The chemicals were supplied by Ralin (BV, Lijinbaan, Netherlands). The study used different microbiome-targeted therapies including Flora Ultra Strength (FUS) probiotic mixture, INNATE Response Formulas (Manchester, USA), Inulin Greena (INU) prebiotic, (RESIPI PHARMA, Cairo, Egypt), Mutaflor (Escherichia coli Nissle 1917 strain) (Ardeypharm GmbH, Herdecke, Germany), Kefir milk (Ready-made probiotics containing milk) (Heal Pharmaceutical, Cairo, Egypt) in addition to Rosavin (Aktin Chemicals, Chengdu, Chinaherbal) as an herbal drug.

### Experimental animal design

**Fig. 1:**
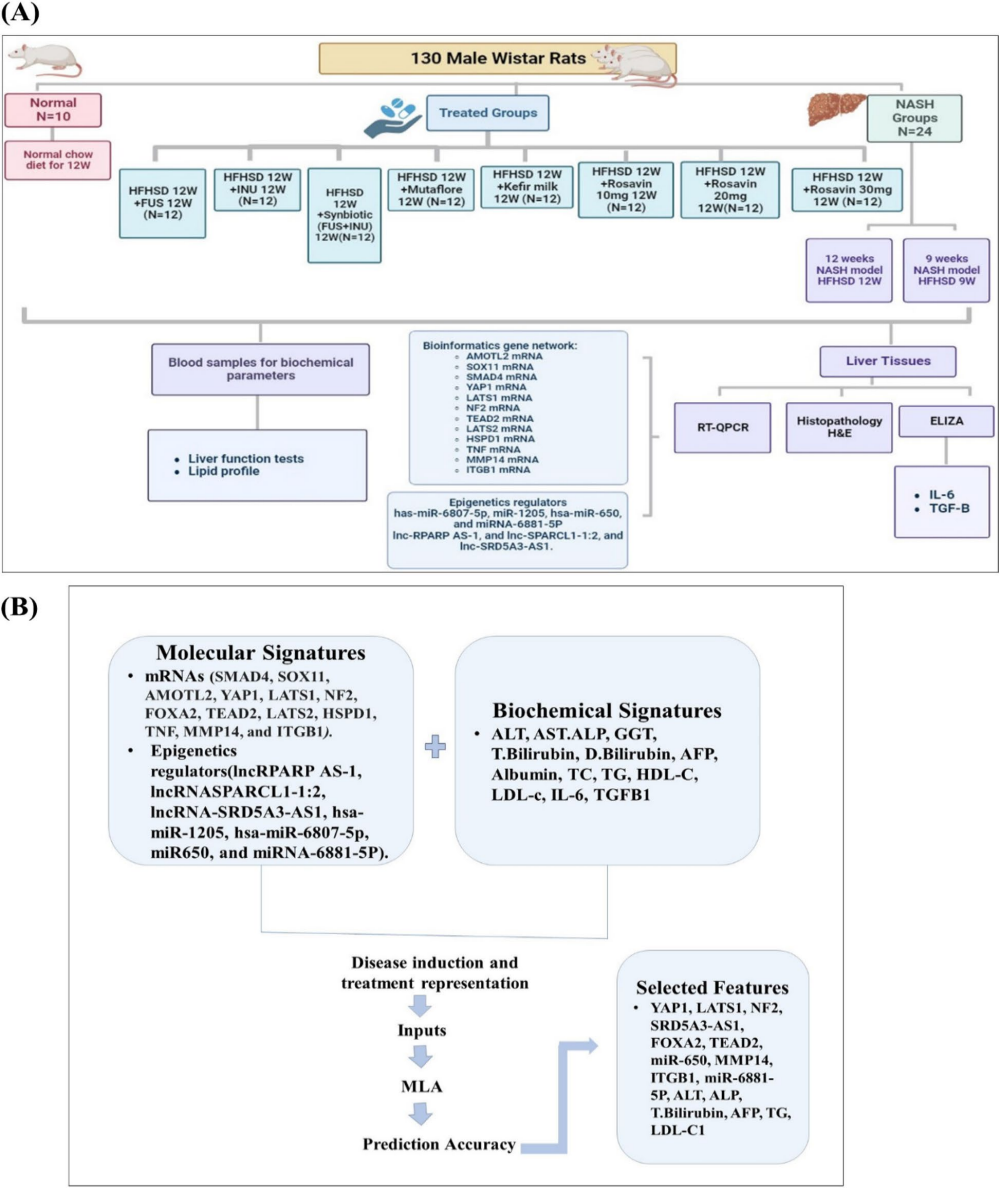
Schematic representation of the experimental protocol. **A** summarizes the study design and laboratory work. **B** illustrates the ML-based model findings

A hundred and thirty male Wistar rats 140–160 g were obtained from Ain Shams University's Scientific Research Centre “MASRI” for this study (Fig. 1A). The rats were maintained in a regulated environment with a 12-h light/dark cycle and a temperature of 20 ± 2℃. Ethical approval (FMASU-R 111/2022, FWA 000017585) was obtained from Ain Shams Faculty of Medicine's Ethics Committee, following NIH guidelines for laboratory animal care and use. Rats were fed a high sucrose and high-fat (HSHF) diet (10% sucrose, 20% fat, supplemented with 68.75% standard chow, 1% cholesterol, and 0.25% bile salts) for 12 weeks to induce NASH [46]. After seven days of acclimating, rats were divided randomly into two groups: standard chow-fed (Normal group, *n* = 10) and high-sucrose/high-fat-fed diet (*n* = 120). The high-sucrose/high-fat-fed rats were subdivided into ten groups (*n* = 12): NASH-12wk (fed the diet for 12 weeks), NASH-9wk (fed the diet for 9 weeks), FUS (administered 4 × 109 CFU bacteria/kg/day of FUS probiotic) [47], INU (administered 2 g/kg/day of INU prebiotic fibers) [48], FUS + INU (administered both FUS probiotic mixtures and INU prebiotic fibers), Mutaflor (administered 4 × 108 CFU of Escherichia coli Nissle 1917/kg bw/day) [49], Kefir (administered 4 × 107 CFU/ml ready-made kefir milk/kg bw/day) [50], and three Rosavin-treated groups (Ros-10, Ros-20, Ros-30) administered with three doses (10, 20, and 30 mg/kg/day) of herbal rosavin treatment [51].

Microbiome-targeted therapies were administered via gastric gavage, while herbal rosavin treatment was administered intraperitoneally, both daily for 12 weeks. Prior to sacrifice, the rats were fasted for 12 h, anesthetized with urethane (1.2 g/kg) intraperitoneally, and blood samples were collected from the retro-orbital vein. The serum was separated by centrifugation (3000 rpm, 10 min, -20°C). Liver tissues were dissected, with one part used for RNA and protein analysis, and the rest fixed in 10% formalin for histopathology and immunohistochemistry. The study design workflow is depicted in Fig. 1.

### Histopathological assessment

Liver samples were fixed in buffered formalin, dehydrated in alcohol, and embedded in paraffin wax. Subsequently, 4-µm-thick sections were prepared from these samples. Hematoxylin and eosin (H&E) staining was employed to evaluate tissue morphology. The severity of NASH was graded on a scale of 0 to 8, where scores of NASH (≥ 5), borderline (3–4), and no NASH (< 3) were assigned [52]. This grading system is based on the sum of scores for steatosis (0–3), lobular inflammation (0–3), and hepatocyte ballooning (0–2) [53].

### Biochemical analysis

The concentrations of multiple serum biomarkers including alanine transaminase (ALT), aspartate transaminase (AST), alkaline phosphatase (ALP), gamma-glutamyl transferase (GGT), total bilirubin (T. Bilirubin), direct bilirubin (D. Bilirubin), albumin, High-density lipoprotein cholesterol (HDL-C), total cholesterol (TC), triglycerides (TG), and Low-density lipoprotein cholesterol (LDL-C) were measured using commercially available kits and an automated Beckman Coulter AU680 autoanalyzer (Beckman Coulter Inc, CA). Alpha-fetoprotein (AFP) levels were quantified using a rat AFP ELISA Kit from MyBioSource (Catalog No. MBS452901, San Diego SDS, CA, USA). Additionally, interleukin-6 (IL-6) and Transforming growth factor beta 1 (TGF-β1) levels in liver tissues were determined with sandwich ELISA kits (catalog numbers: E0079r and E0124R, respectively; EIAab, Wuhan, China) following the manufacturer's instructions.

### RNA extraction and quantification

To extract RNA, liver tissue samples underwent processing with the miRNeasy Mini Kit (Cat. No. 217004, Qiagen, Helman, Germany) following the manufacturer's instructions. The concentration and purity of the isolated RNA were assessed using NanoDrop (Thermo scientific, USA) to ensure a desired purity range of 1.8–2 (A260/A280 ratio). The extracted RNA was then reverse transcribed into complementary DNA (cDNA) using miScript II RT (Cat. No. 218161, Qiagen, Helman, Germany) and RT2 First Strand Kit (Cat. No. 330404, Qiagen, Helman, Germany) with Thermo Hybrid PCR Express (Thermo Fisher Scientific, Massachusetts, USA). The expression levels of *SMAD4, SOX11, AMOTL2, YAP1, LATS1, NF2, FOXA2, TEAD2, LATS2, HSPD1, TNF, MMP14, and ITGB1* mRNAs (Accession: NM_NM_005359.6, NM_003108.4, NR_002819, _001113490.2, NM_001034002, NM_001134543, NM_013193, NM_ 021784, NM_003598, NM_014572, XR_037196, NM_000594, NM_004995, and NM_004763, respectively) were determined. This was done using the Quantitect SYBR Green ROX qPCR Mastermix Kit (Cat. No. 204143, Qiagen, Helman, Germany). The housekeeping gene *GAPDH* and Hs_SNORD72_11 miScript Primers (Accession: QT00079247 and MS00033719) were used to normalize the raw data and compare it with a control sample. The qPCR protocol commenced with an initial activation phase at 95°C for 10 min. This was followed by 40 cycles, each involving denaturation at 94°C for 15 s, annealing at 55°C for 30 s, and extension at 72°C for 30 s. In addition, the expression levels of lncRPARP AS-1, lncRNASPARCL1-1:2, and lncRNA-SRD5A3-AS1 (Accession: ENST00000473970, ENST00000506480, and ENST00000433175) were quantified using the RT2 SYBR Green ROX qPCR Master Mix (Cat. No. 330500, Qiagen, Helman, Germany). The qPCR procedure started with an initial activation step at 95°C for 2 min, then 40 cycles consisting of denaturation at 95°C for 5 s, and combined annealing/extension at 60°C for 10 s. To evaluate the hsa-miR-1205, hsa-miR-6807-5p, miR650, and miRNA-6881-5P (Accession: MIMAT0005869, MIMAT0027514, MIMAT0003320, and MS00048069) expression levels, the miScript SYBR Green PCR Kit (Cat. No. 218073, Qiagen, Helman, Germany) was employed. The qPCR program for this analysis began with an initial activation step at 95°C for 15 min, then denaturation at 94°C for 15 s for 40 cycles, annealing at 55°C for 30 s, and extension at 70°C for 30 s. Real-time quantitative PCR (RT-qPCR) was performed using an Applied Biosystems 7500 FAST RT-PCR system (Applied Biosystems, Foster City, CA, USA) thermal cycler. Duplicate reactions were conducted for each sample, and threshold cycle (Ct) values above 36 were considered negative expressions. The Livak method, RQ = 2^(−ΔΔCt)^, was employed to determine the relative quantification of RNA expression (Appendix Table A.2) [54].

### Statistical analysis

The presented data was expressed as mean ± standard deviation (SD). The normality of data distribution was assessed using the Shapiro–Wilk test. To assess statistical differences among the experimental groups, GraphPad Prism software (version 8.0, San Diego, CA, USA) was utilized to conduct a one-way analysis of variance (ANOVA). Intergroup comparisons were performed using Tukey's post hoc test. Statistical significance was considered when the *p*-value was below 0.05. For the analysis of categorical data, the Chi-square test was utilized.

### Machine learning model building

**Fig. 2:**
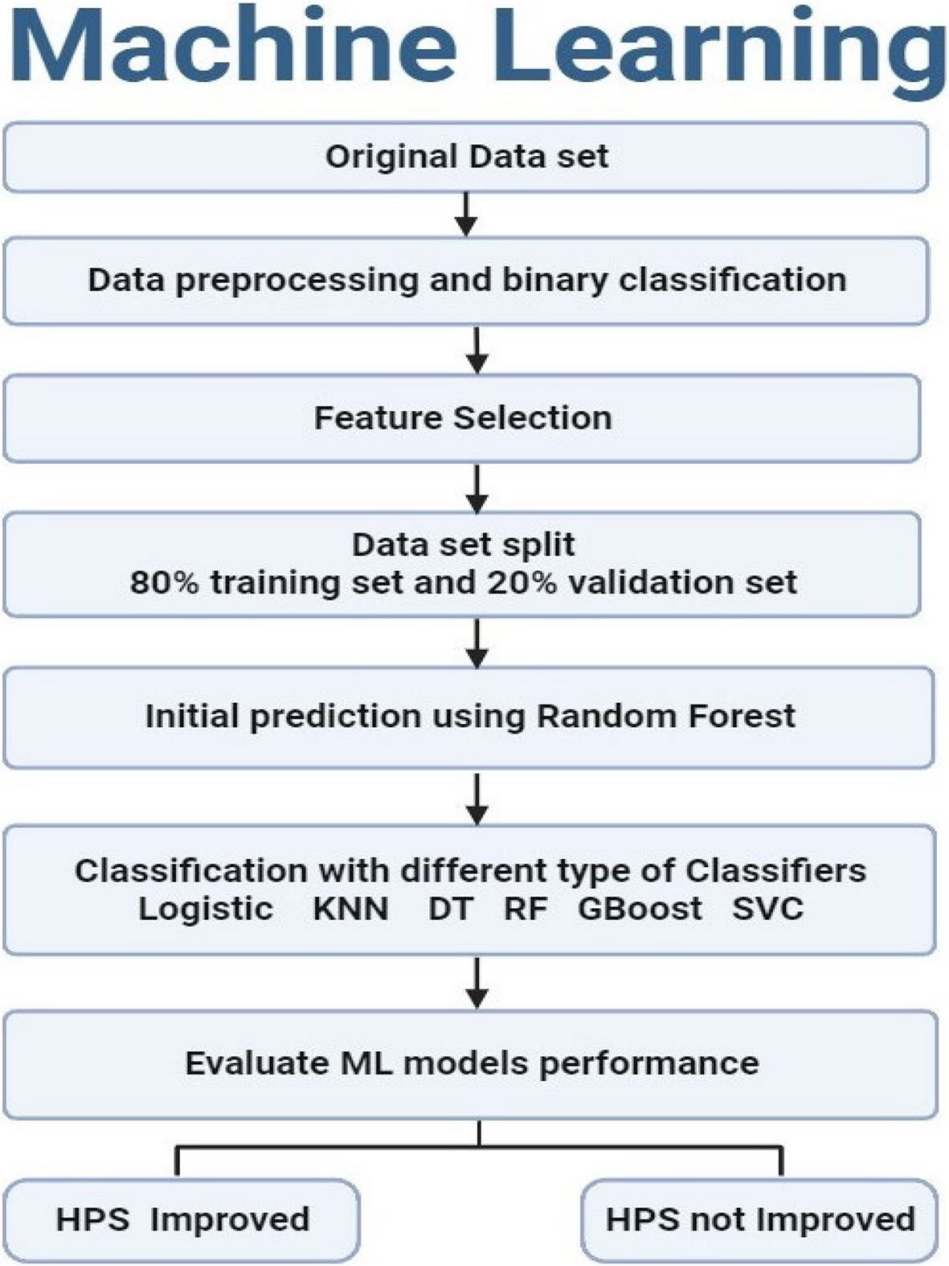
Sequential Steps of the ML Algorithms Illustrated in a Flowchart

In this study (Fig. 2), a comprehensive ML experiment was conducted utilizing a dataset comprising 130 samples with 2 different groups of signatures. The first group consisted of 20 molecular signature features (*HSPD1*, *TNF*, *MMP14*, *ITGB1*, *YAP1*, *LATS1*, *NF2*, *FOXA2*, *TEAD2*, *LATS2*, *SOX11*, *SMAD4*, *AMOTL2*, miRNA-6881-5P,miR-1205, miR-650,miR-6807-5p, lnc-SPARCL1-1:2, lncRNA SRD5A3-AS1, LncRNA RPARP AS-1), while the second group contained 14 biochemical features (ALT, AST, ALP, GGT, T. Bilirubin, D. Bilirubin, AFP, Albumin, TC, TG, HDL-C, LDL-c, IL-6, TGFB1).

The analysis began by conducting machine learning predictions individually for each group of features (Molecular and Biochemical) which allowed us to identify the best features within each group separately utilizing the robust Random Forest algorithm. Then, for the combined features six ML models were used as follows: Random Forest (RF), Logistic Regression (LR), K-nearest neighbor (KNN), Gradient Boosting, Decision tree (DT), and linear support vector machines (SVMs) to comprehensively identify the potential set of features that are highly predictive for the drug response with high accuracy compared to the invasive histopathological interventions, creating a total of three different modeling phases. was split into two sections: 80% was allocated for training purposes, while the remaining 20% served as a validation set for all trials. Additionally, various methods were utilized to mitigate overfitting, boost efficiency, and enhance the models' generalizability. First, Min–Max scaling was employed to normalize the dataset, adjusting features to a range between 0 and 1 to prevent any single feature from dominating the model. Next, outlier detection was conducted to ensure the dataset lacked significant outliers, preventing anomalous data points from biasing the model training. Lastly, Recursive Feature Elimination with Cross-Validation (RFECV) was implemented to determine the optimal subset of features for predicting NASH.

### Preprocessing of the data

Data preprocessing is one of the central steps in machine learning models’ development, ensuring the data's quality and reliability before it is used by classifiers. In this study, there were three datasets: molecular data, biochemical data, and a combined dataset that combined both of them. In the preprocessing, any features with missing values were eliminated, noisy data were cleaned, categorical features were converted into numbers, and identified and removed outliers. Additionally, a check was conducted to ensure the absence of null features.

### Binary classification

The target label was reshaped and modified (Histopathological NASH score) to have only two main classes. The two classes were selected to reflect how close the score is to the extreme scores, 0 or 8. Scores from 0 to 4 were considered as class 0 (HPS Improved), while scores from 5 to 8 were considered as class 1 (HPS Not Improved). However, for biochemical and molecular signatures combined together, the HPS values were kept. That means an adjusted accurate prediction of the HPS exact level without grouping similar scores (9 HPS levels from 0 to 8) was performed. Also, an additional step was performed to adjust the balance between samples belonging to different HPS levels as used-up sampling to increase the small number of cases in specific HPS levels.

### Feature selection

The feature selection technique was employed to reduce the complexity and dimensionality of the data, thereby enhancing learning efficiency. Also, the model becomes faster and more accurate by choosing only the pertinent features, while improving its predicting skills by reducing noise [55, 56].

### Selecting the important features of ML

A preliminary experiment was conducted to identify the machine learning-selected features for predicting improvement levels. The RFECV method was used, which works by progressively eliminating features and evaluating prediction accuracy. The optimal set of predictive features is chosen as the one that maintains or exceeds the original accuracy obtained with the full feature set. The study presents different accuracy levels for various feature combinations.

### Principal Component Analysis (PCA)

PCA was assessed on sets of features before and after the feature selection phase. The PCA analysis shows how the prediction strength could be when using different sets of features. Dimensionality reduction is a process that involves reducing the number of used features in ML algorithms. This may also be utilized for enhancing the performance and accuracy of ML algorithms. One form is to perform data compression. For instance, transform 3D data into 2D data and remove a feature or dimension. It can also be used for reducing dimensions for efficient data visualization. Dimensionality reduction may also be used to speed up the total duration it takes for other learning algorithms to learn. The usage of dimensionality reduction reduces the amount of training samples and/or the number of features, consequently reducing the training’s running time. However, the compressed data still retains the same information as the uncompressed data.

### Feature correlation for each available data set

The goal of this process was to uncover the relationships between the existing features. A correlation matrix was employed to evaluate the correlation coefficients between different pairs of features. A correlation coefficient near + 1 or -1 signifies a strong positive or negative correlation between the two features, respectively, while a value of 0 indicates no relationship between them [57].

### Multi-class classification

In this research, six ML tools were applied, and their performance was evaluated, RF, LR, KNN, GBOOST, DT, and SVM [58].

### Random Forest classifier (RF)

This ensemble learning technique, utilized for classification (and regression), involves constructing numerous decision trees during training and determining the final class through a voting process among these individual trees [59]. Random forests entail creating a collection of uncorrelated trees using a procedure similar to CART, augmented by randomized node optimization and bagging [60].

One significant benefit of RF is its computational efficiency, which results in improved prediction accuracy without a significant increase in computational demands. Moreover, random forests can effectively handle a substantial number of explanatory variables, even reaching into the thousands [61]. As a result, they are recognized as one of the leading algorithms in current machine-learning practices [62].

### Logistic Regression (LR)

LR is a widely employed mathematical modeling technique commonly utilized for epidemiological datasets within the realm of machine learning. Initially, LR computes probabilities using the logistic function. It proceeds to acquire the coefficients necessary for constructing the LR model and ultimately leverages this model for making predictions [63]. This model falls under the category of generalized linear models and consists of two fundamental components: the linear part, responsible for executing the classification model calculations, and the link function, which delivers the output of these computations [64]. It is valued for its simplicity, interpretability, and efficiency.

### K-Nearest Neighbors (KNN)

The KNN is a non-parametric method used for both classification and regression. This technique identifies the k closest training examples within the feature space. In KNN classification, class membership is determined through a majority vote among an object's nearest neighbors, assigning the object to the most common class among its k closest neighbors. Here, k is a positive integer, usually small. When k = 1, the object is assigned to the class of its single nearest neighbor [65].

### Decision Tree (DT)

In ML, DT serves as a predictive model [66]. A DT is structured like a flowchart, each internal node represents a test on an attribute, each branch shows the result of that test, and each leaf node represents a class label. The decision-making process evaluates all attributes, and the path from the root to a leaf node outlines the classification rules. Essentially, a decision tree acts as an adaptive basis function model [67].

### Gradient Boosting (GBOOST)

Ensemble machine learning technique that builds predictive models by sequentially combining simpler models, typically decision trees, to correct errors made by previous models. It works by optimizing a loss function to find the best model weights, making it highly effective for both regression and classification tasks. GBoost often produces robust and accurate predictions but can require careful tuning to prevent overfitting.

### Support Vector Machine (SVM)

SVM [68] is a technique that maps a feature vector into a higher-dimensional vector space [69]. In this space, an optimal hyperplane is defined to maximize the margin between the hyperplane and the nearest data points on both sides. By increasing the separation between the nearest data points from different classes, the overall classification error is reduced.

### Model training parameter of classifiers

The hyperparameters of the RF model, such as maximum tree depth, minimum sample split, and the number of estimators, were adjusted to obtain the best-performing model (Table 1). The optimization process involved experimenting with various parameter combinations for the specific technique being used, aiming to find the most effective configuration for each dataset.

**Table 1:** Random forest hyperparameter tuning

Parameter	Values
max_depth	4
min_samples_split	3
n_estimators	50
random_state	42

### Machine learning evaluation

The performance of the model was assessed using the test dataset. Statistical metrics, such as accuracy, and a confusion matrix were computed to evaluate the effectiveness of the machine learning algorithms. Additionally, the area under the curve (AUC) was utilized for comparison purposes [58].

## Results

### Histological findings

**Fig. 3:**
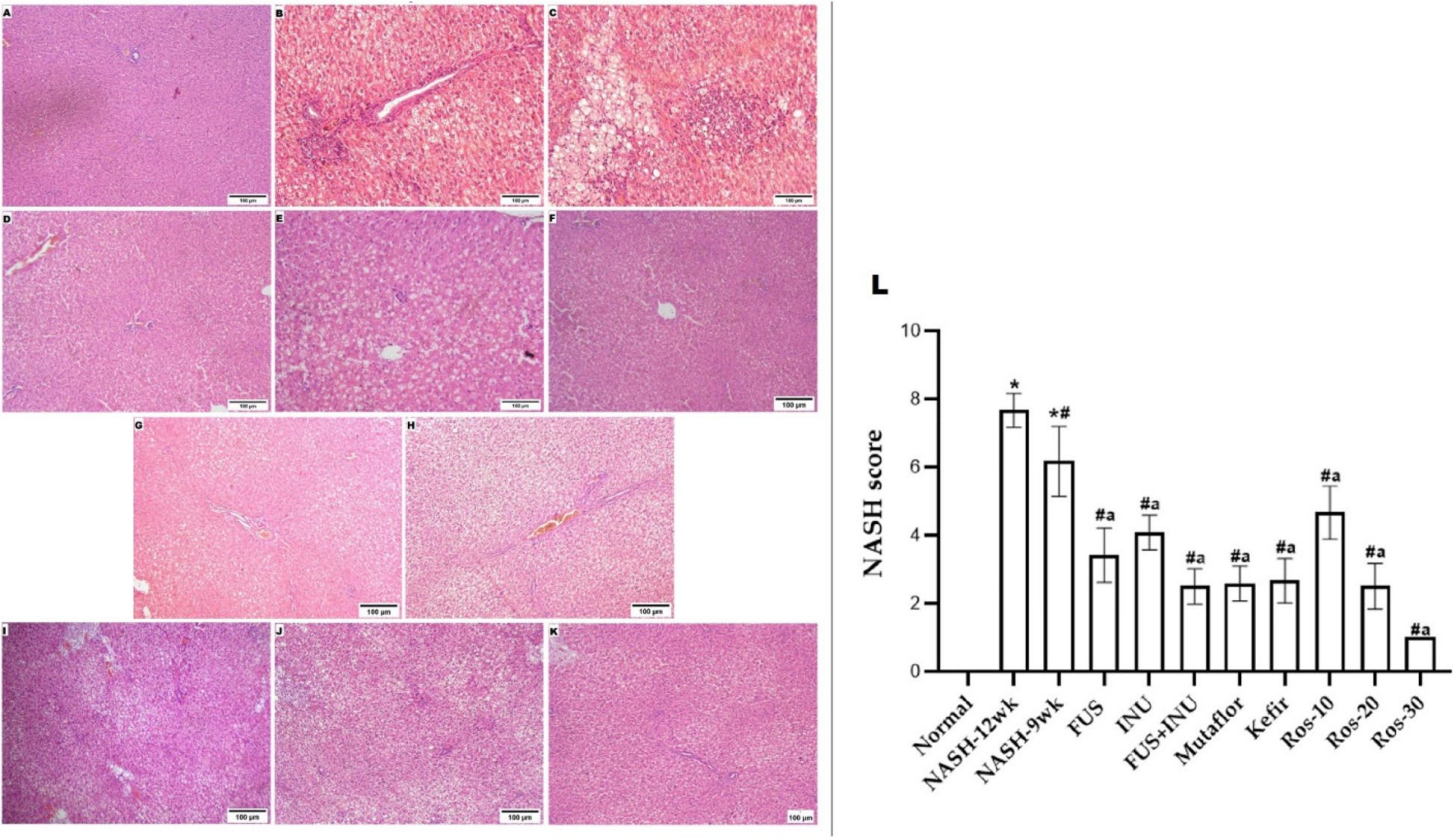
Microbiome-targeted therapies and herbal rosavin impact on hepatic steatosis, inflammation, and fibrosis assessed using H&E staining. Histological examinations revealed (**A**) Normal group: normal architecture. **B** NASH-12 wk and (**C**) NASH-9wk groups: showed severe parenchymal damage, ballooned liver cells, and micro and macro-vesicular steatosis. Additionally, the NASH-12wk group showed substantial fibrosis. Notable histological improvements in steatosis, inflammation, fibrosis, and NASH score in (**D**) FUS, (**E**) INU, (F) FUS + INU, (**G**) Mutaflor, (**H**) Kefir, (**I**) Ros-10, (**J**) Ros-20 and (**K**) Ros-30 treated groups (Magnifications: × 100)

The liver from the normal rats’ group displayed the normal structure of the brownish-red color that has a smooth and shiny morphology without any pathological features. However, livers from the NASH-induced groups or the group treated with herbal rosavin appeared enlarged, yellowish, and harder. Conversely, compared to those of the NASH groups, the livers of rats treated with Microbiome-targeted therapies looked redder and lighter. Histological examination of the livers in the NASH groups revealed severe parenchymal damage, ballooned hepatocytes, and micro and macrovesicular steatosis. Additionally, the NASH-12wk group exhibited substantial fibrosis. While the microbiome-targeted and herbal rosavin-treated groups indicated significant improvements in hepatocyte vacuolation, NASH score, inflammatory response, and fibrosis (Fig. 3) (Appendix Table A.3). Although the improvements in the Ros-10 group were less notable compared to other treated groups, these findings suggest that Microbiome-targeted therapies or herbal rosavin can potentially improve NASH progression. Overall, the group treated with a high dose of rosavin, and the FUS/INU combination group exhibited a significantly greater ameliorative effect compared to other treatments. The groups treated with probiotics (FUS, Mutaflor, and Kefir) and the Ros-20 group showed a similar pattern of improvement.

### Microbiome-targeted therapies and herbal rosavin impact on serum biochemicals

The levels of all serum biomarkers exhibited significant differences among the 11 experimental groups (*P* < 0.001), as shown in Table S1. Rats that were fed an HSHF diet for either 9 or 12 weeks displayed a notable increase in serum liver enzymes (ALT, AST, ALP, albumin, GGT, total bilirubin, direct bilirubin) and lipid profile (TC, TG, and LDL-C), along with elevated levels of the AFP tumor marker. However, HDL-C showed a different behavior and significantly decreased in both NASH groups compared to the group that was normally fed chow. While administration of either microbiome-targeted therapies or herbal rosavin, simultaneously with a high-sucrose/high-fat diet, significantly modulated these variables detected in NASH groups.

### Effect of microbiome-targeted therapies and herbal rosavin on the target effector signatures

Inducing NASH markedly raised the levels of IL-6 and TGF-β proteins in liver tissue compared to the normal group. However, in groups treated with microbiome-targeted therapies and herbal rosavin, there were notable decreases in these protein levels compared to both NASH groups. (*P* < 0.05). These observations suggested that both microbiome-targeted therapies, as well as herbal rosavin, effectively ameliorated the inflammation and fibrosis grades detected in NASH Table S1.

### Effect of microbiome-targeted therapies and herbal rosavin on the expression profile of RNA signatures

**Fig. 4:**
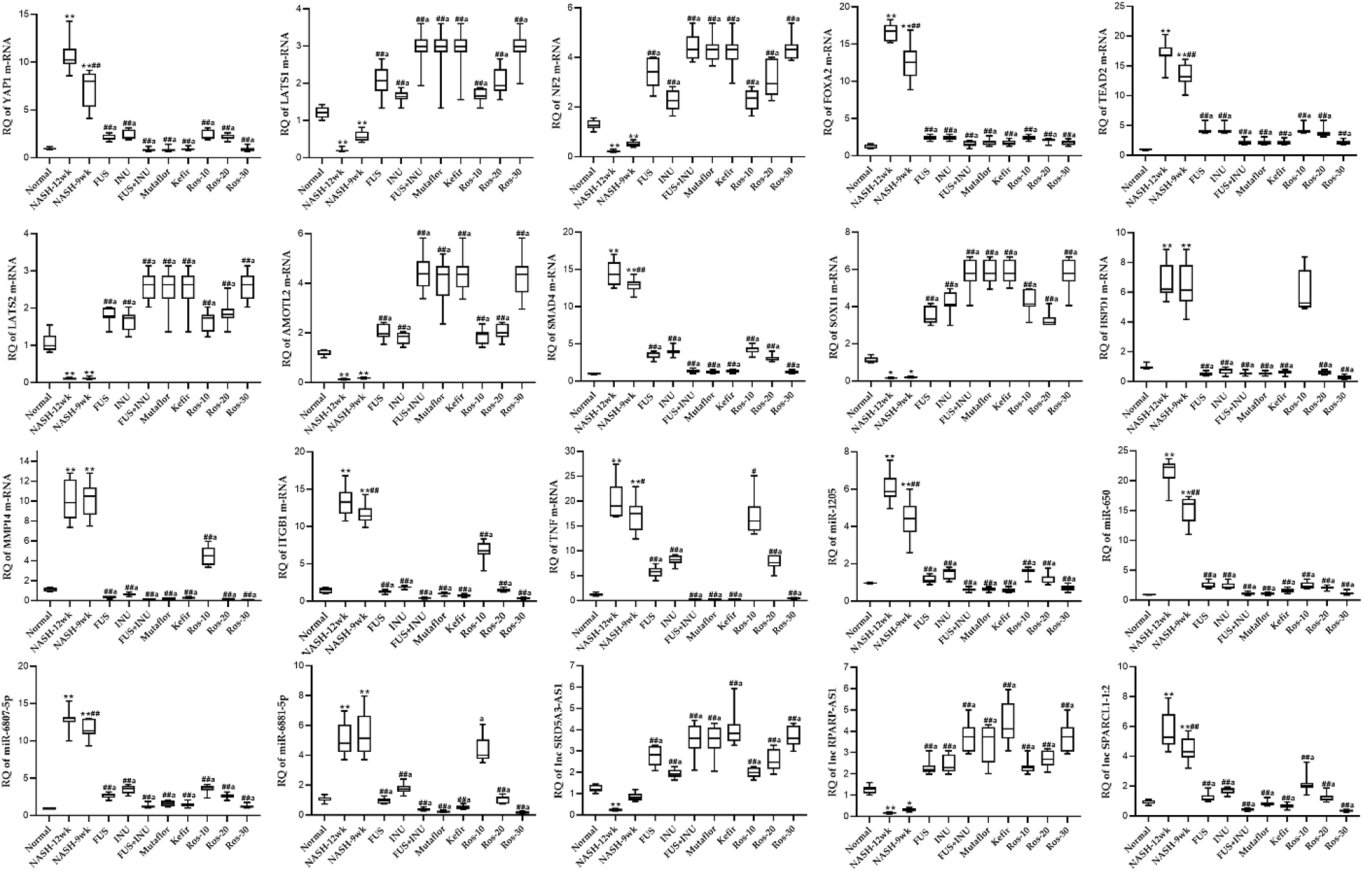
Differential expression of RNA signatures in the hepatic tissues. Values mean ± SD. *P < 0.001 compared to the Normal group. ##*P* < 0.001 and #*P* < 0.05 compared to NASH-12wk group, a*P* < 0.05 compared to NASH-9wk. One-way ANOVA followed by Tukey’s multiple comparison test

Both NASH groups experienced significant increases in the *YAP1, HSPD1, TNF, TEAD2, ITGB1, FOXA2, MMP14*, and *SMAD4*, as well as miR-1205, miR-6881-5p, miR-650, miR-6807-5p, and SPARCL1-1:2 expression. At the same time, *LATS1, LATS2, AMOTL2, SOX11, NF2*, LncRNAs-RPARP-AS1, and SRD5A3-AS1 were significantly suppressed in both NASH groups, in comparison to the Normal group. However, the daily treatment with either microbiome-targeted therapies or herbal rosavin slightly normalized the significant expression of hepatic RNA signatures (Fig. 4). The pronounced effect was observed in the ros-30 treated group and the FUS/INU combination group.

### The ML results

#### Correlation analysis

**Fig. 5:**
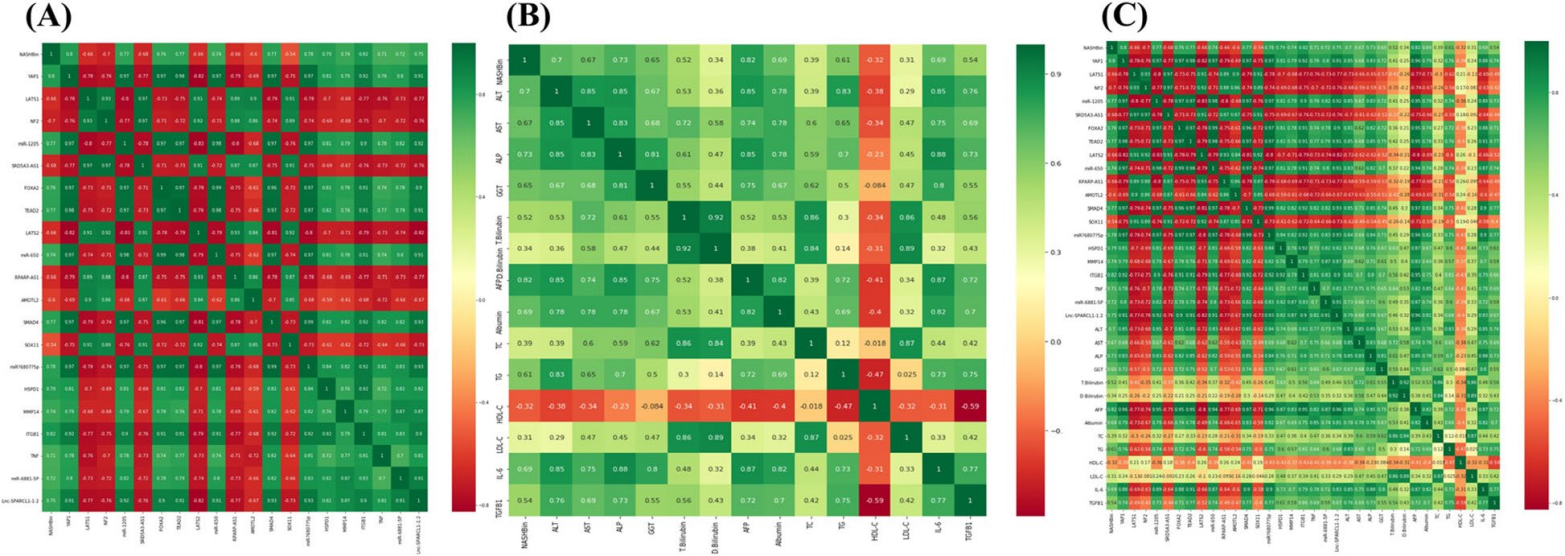
Heatmaps represent the correlation analysis. **A** Correlation matrix for the Molecular features. **B** Correlation matrix for the Biochemical features. **C** Correlation matrix for the combined features

The correlation matrix, as illustrated in Fig. 5, displayed the levels of correlation between pairs of features over the entire dataset for the molecular, biochemical, and both of them combined, providing insight into feature relationships. The correlation coefficient was contained in each cell of the matrix. Based on the connection with the target group feature, Tables 2 and 3 displayed the sorted features that had a high association.

**Table 2:** Sorted molecular features based on correlation with the target group feature

**Molecular Features**	**Correlation Degree**
ITGB1	0.82
YAP1	0.8
HSPD1	0.79
miR-6807-5p	0.78
miR-1205	0.77
TEAD2	0.77
SMAD4	0.77
FOXA2	0.76
Lnc-SPARCL1-1:2	0.74
MMP14	0.74
miR-650	0.74
Mir-9881-5p	0.72
TNF	0.71
NF2	0.7
LATS1	0.66
LATS2	0.66
RPARP-AS1	0.66
SRD5A3-AS1	0.68
AMOTL2	0.6
SOX11	0.54

**Table 3:** Sorted molecular features based on correlation with the target group feature

Biochemical Features	Correlation Degree
AFP	0.82
ALP	0.73
ALT	0.7
Albumin	0.69
IL-6	0.69
AST	0.67
GGT	0.65
TG	0.61
T. bilirubin	0.54
TGFB1	0.54
TC	0.39
D. bilirubin	0.34
HDL-C	0.32
LDL-C	0.31

#### Feature selection and PCA

**Fig. 6:**
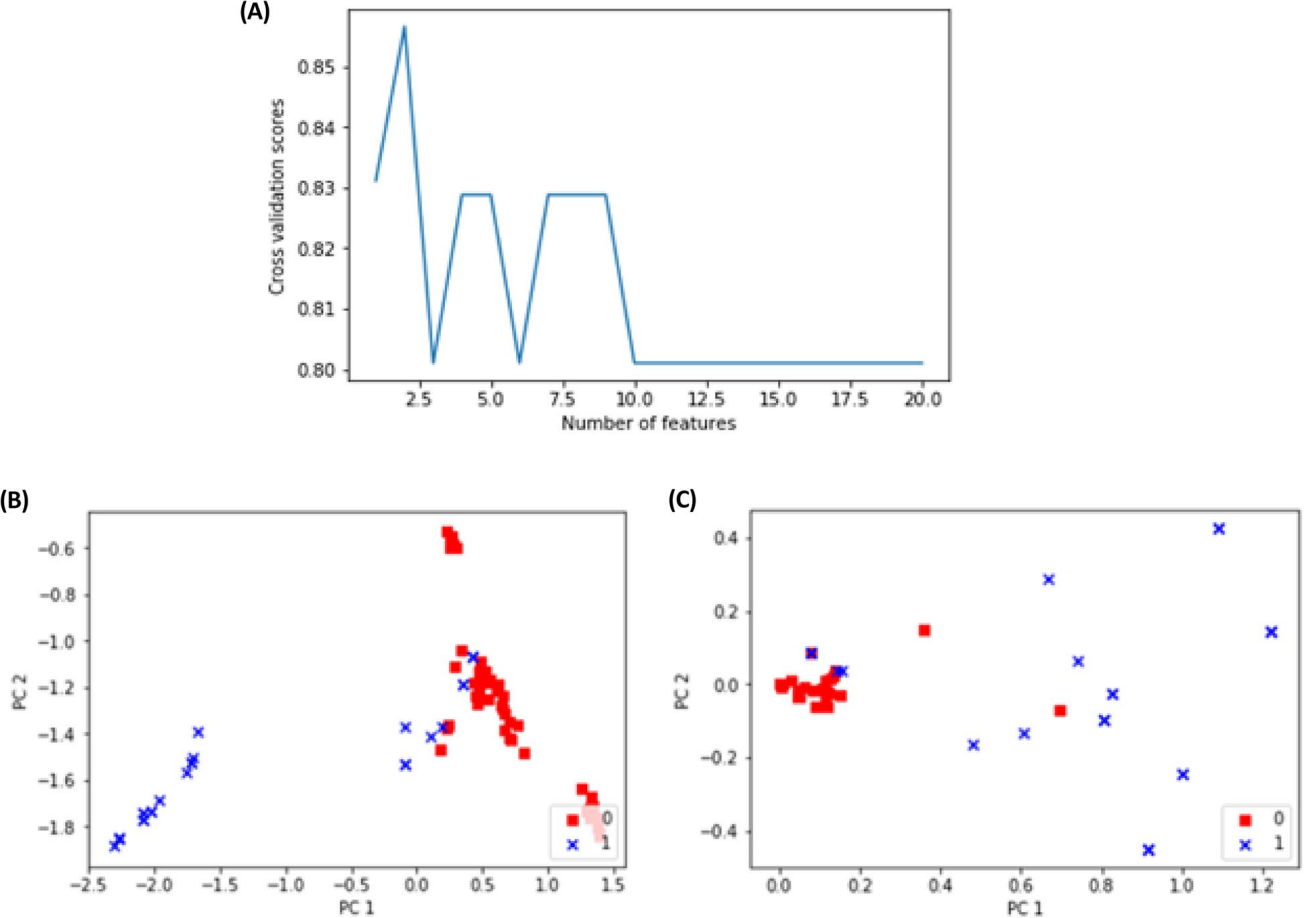
**A** Prediction performance for different numbers of features. PCA analysis for the features **B** before feature selection with 20 features, and **C** after feature selection (2 features)

For the Molecular signatures, the RFECV was adopted [70]. The method selected 2 out of 20 features which kept the same prediction accuracy level. Figure 6A depicts the performance of distinct features. The two selected features are HSPD1 and miR‑6807‑5p. Feature selection phases identified these 2 genes as being the two most important genes in the prediction of improvement. PCA was performed on sets of features before and after the feature selection phase. The PCA analysis shows how the prediction strength could be when using distinct sets of features. The set of selected features for the molecular signatures could keep the same level of prediction accuracy although 18 features were dropped. The selected set of features showed good discrimination between the two reconstructed categories of Improved case scores when examined by the PCA plot as shown in Fig. 6B&C.

**Fig. 7:**
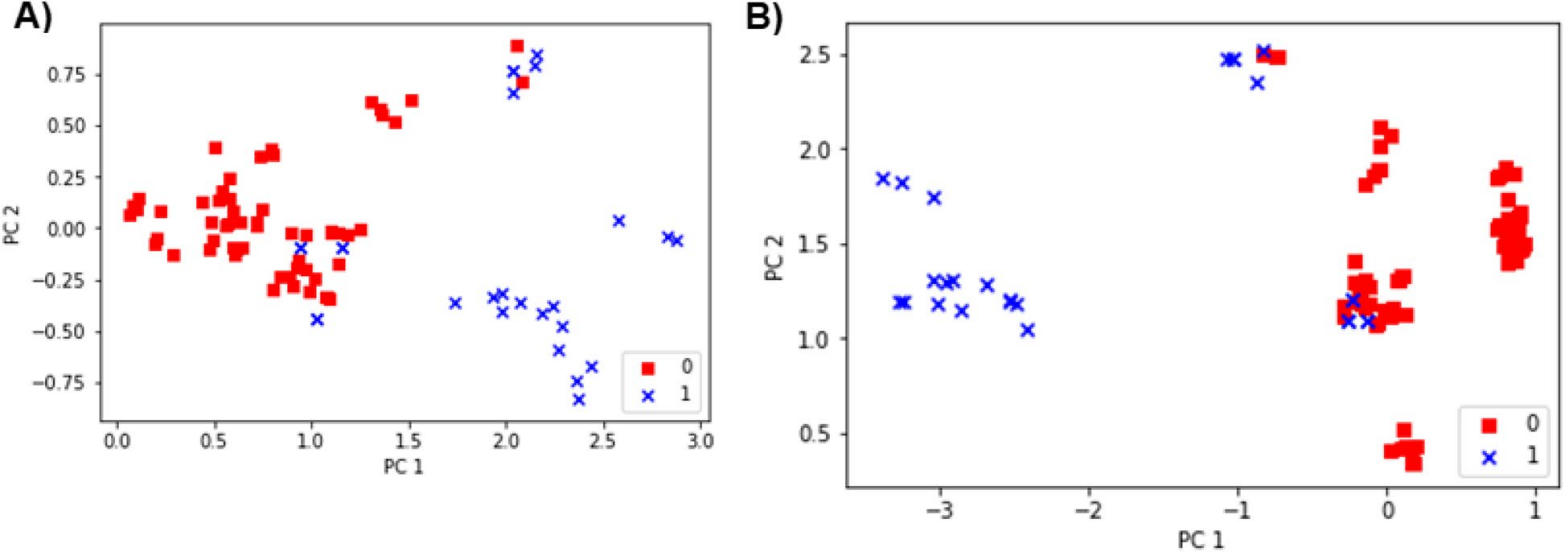
**A** PCA analysis for the biochemical features, **B** PCA analysis for the combined features

However, the feature selection for the Biochemical Signatures did not show any improvement when features were reduced. Thus, the whole set of 14 features is used for predictions as they could together differentiate between predicted classes. The discriminative ability of the features is shown in the PCA (Fig. 7A). Also, the discriminative ability of the combined 34 features is shown in the following PCA (Fig. 7B).

#### Confusion matrix of random forest classifier

The confusion matrix presented in Table 4 shows the correctness of the predictions for both classes, improved (HPS: 0 → 4) and Not-Improved (HPS: 5 → 8) based on the molecular data only. In the test set results, the Improved samples that were predicted correctly were 18 out of 20), while the Not-Improved samples were all correctly predicted (6 out of 6) based on the molecular data only. The correct portions of prediction are highlighted in bold in the above confusion matrix. While the confusion matrix presented in Table 5 shows the correctness of the predictions for both classes, improved (HPS: 0 → 4) and Not-Improved (HPS: 5 → 8) based on the biochemical data. In the test set results, the Improved samples that were predicted correctly were 20 out of 20), while the Not-Improved samples were all correctly predicted (6 out of 6). The correct portions of prediction are highlighted in bold in the above confusion matrix. Finally, the correctness of the predictions for both classes is presented in Table 6, Improved (HPS: 0 → 4) and Not-Improved (HPS: 5 → 8) based on both the molecular and biochemical features. In the test set results, the Improved samples that were predicted correctly are (20 out of 20), while the Not-Improved samples were all correctly predicted (6 out of 6). The correct portions of prediction are highlighted in bold in the above confusion matrix.

**Table 4:** Confusion matrix for the molecular data

**Actual**	**Predicted**	
	**Improved**	**Not-Improved**
	18	2
	0	6

**Table 5:** Confusion matrix for the molecular data

**Actual**	**Predicted**	
	**Improved**	**Not-Improved**
	20	0
	0	6

**Table 6:** Confusion matrix for both the molecular and biochemical data

**Actual**	**Predicted**	
	**Improved**	**Not-Improved**
	20	0
	0	6

#### Area Under the Curve (AUC)

**Fig. 8:**
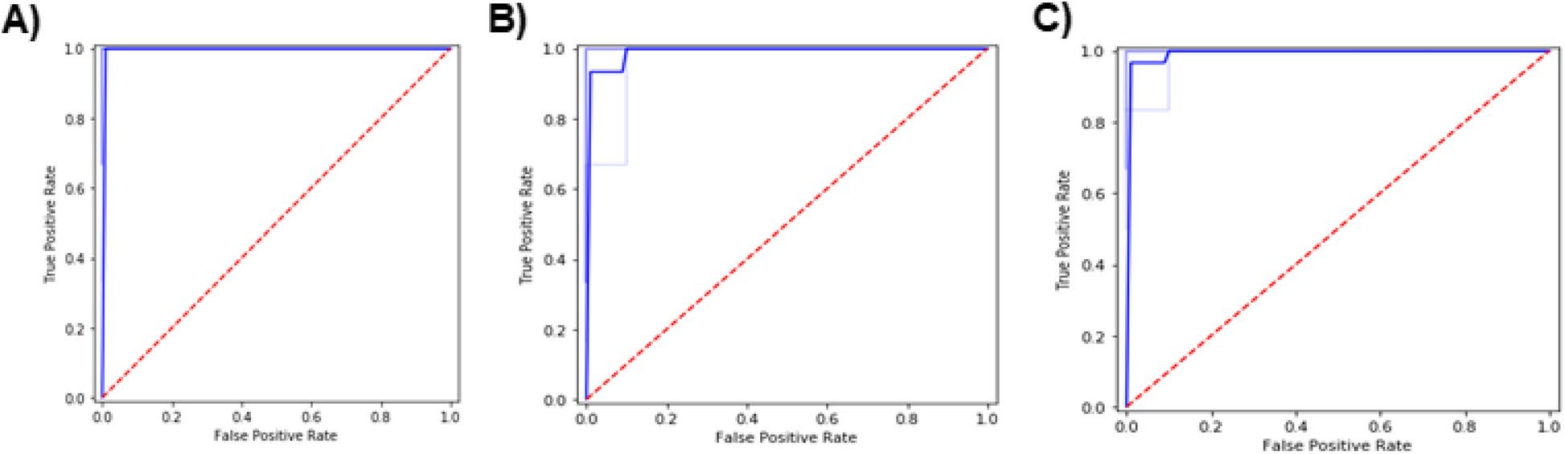
**A** AUC for the Molecular features. **B** AUC for the Biochemical features. **C** AUC for the Combined features

For the molecular features, the cross-validation results for both feature sets showed almost identical results. Also, the average area under the ROC curve was very high with the selected feature set predictions. Figure 8 shows the comparison between the full and selected feature set models when plotted on the AUC plot, displaying how accurate the prediction models were. Only a few numbers of instances were misclassified. The average accuracy achieved was 98%. The True positives refer to the improved samples that were predicted correctly. While the True negatives refer to the Not Improved samples that were correctly predicted. On the other hand, the cross-validation results of the biochemical features showed that the average accuracy achieved was 97%. The average AUC was 99% (Fig. 8). Additionally, the cross-validation results of both the molecular and the biochemical features showed that the average accuracy achieved was 98%. The average AUC was 100% (Fig. 8).

### Multi-class classification and performance evaluation results

The prime incentive of the multi-class classification was the development of models capable of distinguishing between the two category classes (HPS Improved, class 0), (HPS Not-Improved, class 1). The 2 main columns show two different up-sampling stages. Initially, the results of the GBoost Classifier outperformed Random Forest, DT, SVC, LR, and KNN algorithms. The performance of the models is summarized in Table S2. After up-sampling, the results of the machine learning models applied to our dataset focused on the molecular features only showed that the GBoost model stands out as the top performer, achieving an impressive accuracy of 94.91% and a test accuracy of 94.44% reflecting its solid scores with the molecular data. Furthermore, the model exhibits great precision at 0.95 which evaluates the model's ability to reduce false positives and recall at 0.94 which shows how many of the true positive cases were successfully predicted. For the biochemical features, the Support Vector Machine with Logistic Regression (SVC-LR) model emerges as the standout performer achieving an accuracy of 89.35% and a test accuracy of 90.74%. Also, scoring a decent precision of 0.94 and recall of 0.92. For the combined features the GBoost model outperformed other algorithms, achieving the highest accuracy at 99.07% and test accuracy at 98.15% as well as for precision and recall scoring 0.98. The confusion matrix presented in Fig. S1 shows the correctness of the predictions for all classes, starting from class 0 up to class 8,

### Selecting the most important features

In this experiment, the feature selection was performed with many details and using different techniques. The methodology of feature selection was based on gradual decrement of features and continuous checking for prediction accuracy. At some point, the accuracy will drop by a significant value. The set of features before this drop is considered the best prediction feature set. The selected set of molecular features were *YAP1, LATS1, NF2, SRD5A3-AS1, FOXA2, TEAD2,* miR-650*, MMP14, ITGB1*, miR-6881-5P, while the biochemical were ALT, ALP, T. Bilirubin, AFP, TG, LDL-C. Using these features only resulted in a highly accurate prediction using the RF technique. The prediction accuracy was 98.4% (Fig. S2).

## Discussion

Despite the availability of several scoring systems for NASH severity classifications such as the NASH Clinical Research Network (NASH CRN) and the NAFLD Activity Score (NAS) that categorize NASH based on fibrosis stages or multifactorial scores including steatosis, cellular ballooning, and inflammation. These systems have limitations in capturing the full spectrum of cellular and stromal damage that drives the disease, and they are not sensitive enough to accurately indicate changes in the severity of the disease over time. Additionally, the currently available scoring systems illustrate only moderate-to-fair reproducibility [52]. Despite these drawbacks, the current framework for approving NASH therapies depends on pathological scoring methods performed manually [71, 72].

Intestinal microbes play a crucial role in metabolizing dietary compounds and producing bioactive molecules, impacting both physiological and pathological conditions that have the potential to promote HCCs and chronic liver diseases’ development and progression [73]. Accordingly, targeting the intestinal permeability as well as gut microbiome through interventions like fecal microbiota transplantation, diet, synbiotics, probiotics, and prebiotics shows promise as one of the innovative approaches for MAFLD/NASH treatment [74]. Animal studies have shown that probiotics have a profound effect on NASH, decreasing fatty acid production, metabolic endotoxemia, and inflammation. Wagnerberger et al. [75] proved that the administration of *Lactobacillus casei* strain *Shirota* probiotic are found to mitigate fructose-driven NAFLD by attenuating the hepatic Toll-like receptor 4 signaling cascade. A few of the research works have reported improvement in liver functions and histology with probiotic treatments, but additional randomized controlled trials are required to confirm their therapeutic effectiveness [76].

Recently, numerous research works have diagnosed MAFLD and/or NASH using ML focusing on particular avenues of the disease such as quantifying lipid content, staging by imaging, fibrotic status, or even using clinical practice records [77, 78]. Moreover, integrating ML algorithms into established bioinformatics could present tools that accurately predict or assist in the diagnosis of NASH, and identify therapeutic targets [79]. Sorino et al. [80] developed a web application on the basis of a Neural Network (NN) that utilized medical records to predict NAFLD, especially its absence. The application demonstrated a high level of accuracy of 77.0%, specificity of 100%, and sensitivity of 73%.

Therefore, the present research aims to use bioinformatics and ML techniques to develop a reliable model to predict drug efficacy in NASH. To achieve this, the previously established RNA-biochemical signatures were re-validated on a larger sample size of animals across different NASH therapy modules, involving microbiome-targeted therapy and herbal drugs. Our goal is to present a group of features that can assist in predicting the response to different therapeutic modalities for NASH. Herein, various public microarray databases were utilized for selecting functionally linked RNAs and biochemical signatures involved in pathogenic mechanisms driving the NASH pathogenesis, at genetic, epigenetic, and proteomic levels. Consequently, mRNAs (*TEAD2, YAP1, LATS1, LATS2, NF2, FOXA2, AMOTL2, SOX11, SMAD4, MMP14, HSPD1, ITGB1, TNF*), miRNAs (miR-1205, miR-6881-5p, miR-650, miR-6807-5p), lncRNAs (RPARP-AS1, SPARCL1-1:2, and SRD5A3-AS1) and target effectors (IL-6 and TGF-β1) were retrieved in addition to the serum liver enzymes and lipid profile biomarkers (ALT, AST, ALP, Alb, GGT, T. Bilirubin, D. Bilirubin, TC, TG, and LDL-C) and the AFP.

In the current study, the treatment of animals with either microbiome-targeted therapies or herbal rosavin for 12 weeks diminished the histopathological and metabolic abnormalities induced in the NASH groups. They decreased the degree of NASH score, the hepatic level of IL-6 and TGF-β1 as well as improvement in both liver biomarkers and lipid panel. The hepatoprotective effect of either microbiome-targeted therapies or herbal rosavin may be attributed to diminished pro-inflammatory cytokines’ production, inhibition of inflammatory signaling, antioxidant signaling pathways’ activation, enhanced cellular immunity, and decreased tissue apoptosis [34, 36].

Regarding mRNA signatures, there were significant increases in the hepatic expression of *YAP1, HSPD1, TNF, TEAD2, ITGB1, FOXA2, MMP14,* and *SMAD4*, with significant decreases in *LATS1*, *LATS2, AMOTL2, SOX11*, and *NF2* in both NASH groups, as against the Normal group. The daily treatment with either microbiome-targeted therapies or herbal rosavin modulated the expression of hepatic mRNA signatures by ameliorating the signaling pathways contributing to NASH progression including Hippo signaling, TGF-β signaling, TNF signaling, and apoptosis. The Hippo signaling pathway is a highly conserved regulatory system that controls the hepatic size, liver regeneration, and hepatic cell differentiation and survival [81]. *YAP* serves as a pivotal downstream mediator of the Hippo signaling pathway. Upon dephosphorylation, it translocates to the nucleus where it forms a complex with the transcription factor *TEAD*, facilitating essential cell proliferation during liver regeneration. However, prolonged activation of *YAP* can lead to the development of malignant tumors. *NF2* and angiomotin (*AMOT*), situated upstream in the Hippo pathway, contribute to *YAP* phosphorylation via *LATS1/2* during cell fate determination and developmental processes. *SMAD4*, a member of the SMAD protein family, exhibits dual roles as both a tumor suppressor and an oncogene in cancers such as hepatocellular carcinoma. *FOXA2* expressed prominently during early fetal liver development but absent in mature hepatocytes or bile duct cells, represents a potential marker for hepatic cell proliferation [81]. TGF-β signaling mechanism performs a prominent part in the maintenance of normal homeostasis of the liver and is involved in different stages of liver fibrosis, which further contributes to the progression of NAS [82]. TNF signaling is one of the primary regulators of inflammation. It has decisive functions in tissue homeostasis, regulating cell death, and inflammation, which suggests a potential interlinkage between the severity of NASH and its distinct levels [83]. Evidence shows that activated Kupffer cells can worsen liver injury by activating TGF-β and TNF signaling, leading to the production of new extracellular matrix (ECM) proteins like collagen, which contribute to liver fibrosis. This process activates matrix metalloproteinases (MMPs) such as MMP14, which induce the expression of hepatic vascular adhesion molecules. Activated integrins, such as *ITGB1*, on immune cells interact with adhesion molecules to promote cell detachment from the ECM. Dying hepatocytes release stress signals that affect nearby hepatic cells, triggering inflammation and fibrosis [36]. Therefore, the modulatory effect of microbiome-targeted therapies or herbal rosavin on mRNA signatures may result from inhibition of the Hippo, TGF, and TNF signaling pathways, and activation of antioxidant signaling pathways. In the current study, the combination of probiotics and prebiotics is more ameliorative than administering them individually because prebiotics serve as a food source for probiotics, enhancing their growth and activity. This synergistic effect can lead to a more robust and effective modulation of the gut microbiome, resulting in improved NASH progression. Furthermore, the combination group and the high-dose rosavin-treated group exhibited a similar modulatory effect. This may be owing to their action on similar pathways or mechanisms.

In the current study, a 12-week duration was chosen based on several considerations, including previous studies, the nature of the experimental model, and practical constraints. Many studies have successfully employed a 12-week duration to evaluate the efficacy of treatments in similar experimental models of metabolic disorders [84–87] finding this period sufficient to observe significant physiological and metabolic changes and providing meaningful insights into the immediate effectiveness of the therapies. Additionally, the treatments applied in our study have previously been tested in human subjects, showing prospective effects and demonstrating safety and efficacy over extended periods in clinical settings (Table S3). These prior human studies provide a strong foundation for our choice of treatments.

Additionally, the outcomes revealed a significant upsurge in the hepatic expression of miR-1205, miR-6881-5p, miR-650, miR-6807-5p, and SPARCL1-1:2 in the two NASH groups (NASH-12wk and NASH-9wk) compared to the Normal group. Moreover, there was a notable decrease in the levels. The expression of these miRNA and lncRNA signatures has been reversed with microbiome-targeted and herbal rosavin therapies, along with diet manipulation. The functional enrichment of these epigenetic signatures showed their involvement in NASH-related pathogenic mechanisms including TGF-β and Hippo signaling. This can explain their differential expression level among the NASH groups and the other treated groups. The important part of miRNAs and lncRNAs in developing NASH are widely recognized. miRNAs regulate gene expression by binding to specific mRNA sequences, resulting in either the degradation of mRNA or the suppression of translation [88]. Meanwhile, lncRNAs act as miRNA sponges, preventing the degradation of mRNAs targeted by miRNAs, thereby preserving their stability [89]. These molecules are involved in the progression of NASH [90], owing to their strong association with the inflammatory and fibrotic mechanisms and pathways that drive the disease pathogenesis [91]. Herein, different machine-learning models; (RF, LR, KNN, GBoost, DT, and SVM) were used to build models that can differentiate between our two classes (HPS Improved), or not (HPS Not-Improved). The data was sectioned into two partitions: 80% for training and 20% for validation. Also, adjustments were made to balance samples belonging to different HPS levels by using up sampling to increase the number of cases in specific HPS levels. The model's effectiveness was assessed using various performance evaluation metrics, including accuracy, precision, and recall, in addition to the analysis of the confusion matrix. For the combined molecular and biochemical features, GBoost was the best model outperforming other algorithms in terms of all evaluation metrics achieving an accuracy of 98%. However, after a gradual decrement of features, the results showed a better performance when using the Random Forest classifier, achieving an accuracy of 98.4%. The top selected set of molecular features was *YAP1*, *LATS1*, *NF2*, SRD5A3-AS1, *FOXA2*, *TEAD2*, miR-650, MMP14, *ITGB1*, miR-6881-5P, while the biochemicals were ALT, ALP, T. Bilirubin, AFP, TG, LDL-C. This variation of the result may suggest the dynamic nature of machine learning model selection and its sensitivity to feature composition [92]. The principal component analysis (PCA) before and after the feature selection phase showed that the 9 selected features could keep the same level of prediction accuracy and showed better discrimination between classes (NASH vs. Non-NASH). Ultimately, the RF classifier containing 9 features showed very high accuracy and the average area under the ROC curve with the selected 9 features was 0.99 which means that the 9 features have superior diagnostic characteristics for NASH disease **(**Fig. 1B**)**.

In this study, different machine learning methods were tried to compare performance over our dataset. Iterative experiments with optimizations showed that Random Forest is performing well over other methods. The set of algorithms was selected based on either the simplicity or interpretability of the results. In addition, other methods were used for comparison and evaluation of performance.

The process began with Decision Tree methods, which highlight important features that achieve higher information gain during the splitting of tree nodes. Decision Trees are easily interpretable models able to capture non-linear relationships and feature importance. Random Forest provides the same ability while being more resistant to overfitting by using an ensemble of trees. Knowing the important features is crucial to help reduce experimental efforts. RF handles high-dimensional data and is robust to outliers and noisy data.

Other methods used for comparing performance included: Logistic Regression K-Nearest Neighbors (KNN), SVM, and lastly Gradient Boosting which is an ensemble method that sequentially builds models to correct the mistakes of previous models and achieves high predictive accuracy. Although it has a high predictive ability, it is more prone to overfitting and less effective in deciding feature importance when compared to Random Forest.

To tackle concerns about overfitting, a multi-step strategy was developed to strengthen our model's ability to generalize effectively. Initially, The data was rigorously preprocessed, cleansing it of any null values or duplicates to ensure integrity and remove potential biases. RFECV was then utilized to identify the most crucial features of the model. This method systematically prunes less relevant features, homing in on those that carry the most discriminatory power and reducing the risk of overfitting. In summary, ensuring the model's generalizability was a priority. The approach encompassed thorough data preprocessing, feature selection via RFECV, and the application of diverse algorithms. These combined efforts are believed to enhance the robustness and reliability of the findings.

AST, ALT, and ALP are typically utilized as indicators of liver injury, but they lack specificity in diagnosing NASH [11, 93]. Ma et al. [94] developed a machine learning predictive model for NAFLD, by selecting 5 features such as weight, TG levels, ALT, GGT, and serum uric acid levels that provided the most accurate predictions in their Bayesian network model achieving an accuracy rate of 83%, a specificity of 0.878, and a sensitivity of 0.675. By leveraging extensive electronic health records from the United States, Fialoke et al. [95] established a machine-learning model that was capable of accurately predicting NASH. This model incorporated longitudinal data of key biomarkers such as AST, ALT, AST/ALT ratio, and platelet count, along with basic demographic information and diabetes status (AUC = 0.88).

Costello et al. [96] emphasized predicting drug response based using genomic, epigenomic, and proteomic datasets collected from human breast cancer cell lines. Ali and Aittokallio [97] proposed a Bayesian multi-task learning model on a dataset of 53 breast cancer cell lines that integrates data from multiple omics sources including RNA-sequencing, point mutations, somatic DNA copy number variation, protein abundance, transcript expression, and DNA methylation to predict drug response reporting that this multi-task learning model allows the model to learn a more inclusive view of the cancer cells and to make more accurate predictions of drug response suggesting the machine learning a promising tool for precision oncology.

Stetson et al. [98] reported three extensive pharmacogenomic investigations, involving the screening of anticancer compounds across more than 1,000 diverse human cancer cell lines. The datasets were combined for creating and authenticating multi-omics predictors for drug responses. The study makes a comparison of drug response models constructed via various techniques, including a support vector machine, penalized linear regression model, as well as random forest. The reliability and accuracy of each drug response model were rigorously evaluated through cross-validation across three independent datasets. Notably, in comparison to the commonly used elastic net regression as well as SVM, the RF yielded more robust and precise prediction signatures. The resulting drug response signatures can be used to stratify patients into treatment groups based on their tumor biology, offering two significant advantages: facilitating the repositioning and repurposing of existing anticancer therapies and expediting the process of bringing preclinical drugs to market.

### Strengths and limitations

The strength of this study lies in its integration of bioinformatics and ML that utilized the biochemical and molecular features to identify reliable NASH drug efficacy predictors. The model was developed using a high-quality, biopsy-confirmed dataset, crucial for accuracy and reliability. By utilizing biopsy-confirmed diagnoses as the gold standard, the study identified relevant non-invasive biomarkers, enhancing the model's predictive power and clinical relevance. Additionally, the inclusion of multiple ML algorithms such as RF, LR, KNN, GBoost, DT, and SVM enhances the robustness of the predictive model. However, this study also has some limitations. While it sheds light on the potential effectiveness of microbiome-targeted therapies and herbal remedies in managing NASH, it's essential to recognize certain drawbacks. It primarily examined a limited selection of such drugs, possibly not fully representing the spectrum of potential interventions for NASH. Future investigations should encompass a broader range of pharmaceutical agents and treatment methods. Future studies will include a wider variety of NASH models, such as genetically modified mice and additional dietary models, to capture an even broader range of disease phenotypes and enhance the generalizability of the findings. Also, models derived from data collected in animal models need comprehensive validation in clinical settings. It is crucial to conduct validation studies across diverse human populations with NASH and real clinical scenarios to validates the effectiveness of the applied treatments as well as to ensure the reliability and generalizability of these models.

## Conclusion

The objective was to develop an algorithm to evaluate the efficacy of conventional liver blood tests combined with a panel of interacting mRNAs-miRNAs-LncRNAs in identifying a specific set of features that can reliably predict drug efficacy in Nonalcoholic Steatohepatitis (NASH). Utilizing noninvasive biomarkers for predicting treatment response reduces the need for invasive procedures like liver biopsies, thereby minimizing patient discomfort and associated risks. This approach can drive the development of new therapeutic targets and strategies, potentially leading to more effective treatments for NASH.

### Supplementary Information

Supplementary Material 1. Fig. A.1. Showing the pathogenic pathways and processes involved in NAFLD/NASH genesis through the KEGG pathway database, Fig. A.2. showing the involvement of the biochemical-RNA signatures in pathogenic mechanisms (Hippo signaling, TGF-β signaling, TNF signaling pathway, apoptosis, oxidative stress, and inflammatory response) through the KEGG pathway database, and GeneCards database; Fig. A.3. Validation that our selected mRNAs are key regulatory genes in gut microbiota, Fig. A.4. Validation of the interaction between the selected *mRNAs *and the retrieved miRNAs from mirwalk3; Fig. A.5. Validation of the relation of the candidate miRNAs to pathogenic mechanisms such as Hippo signaling, and TGF-β signaling through DIANA tools mirPath 3; Fig. A.6. Validation of the interaction between the selected *miRNAs *and the retrieved lncRNAs from mirwalk3 and DIANA-LncBase; Table A.1. The detailed differentially expressed genes in NASH were retrieved from the gene chip datasets GSE164760, GSE24807, and GSE126848, Table A.2. List of primer assays; Table A.3. Histopathological scoring grid for NAFLD/NASH liver sections.

## Data Availability

All data generated during this study are included in this article. The source code for rebuilding the models used in the study can be found at: https://github.com/ibrahimzb/MLModels_NASHAnimal Supplementary Table A4.

## Abbreviations

MDHC: Metabolic dysfunction-related hepatic condition
NAFLA: Non-alcoholic fatty liver ailment
NASH: Nonalcoholic Steatohepatitis
ML: Machine learning
HPS: Histopathological NASH score
RF: Random Forest
LR: Logistic Regression
KNN: K-nearest neighbor
GBOOST: Gradient Boosting
DT: Decision tree
SVM: Support vector machines
T2DM: Type 2 diabetes mellitus
HCC: Hepatocellular carcinoma
AST: Aspartate aminotransferase
ALT: Alanine aminotransferase
NAS: NAFLD Activity Score
DRP: Drug response prediction
RFECV: Recursive Feature Elimination Cross Validation
PCA: Principal Component Analysis
AUC: Area under the curve
SVC-LR: Support Vector Machine with Logistic Regression
YAP1: Yes Associated Protein 1
LATS1: Large tumor suppressor 1
NF2: Neurofibromin 2
LATS2: Large tumor suppressor 2
TEAD2: TEA Domain Transcription Factor 2
FOXA2: Forkhead box protein A2
AMOTL2: Angiomotin Like 2
SOX11: SRY-Box Transcription Factor 11
SMAD4: Mothers Against Decapentaplegic Homolog 4
MMP14: Matrix metalloproteinase 14
HSPD1: Heat shock protein Family D Member 1
ITGB1: Integrin Subunit Beta 1
TNF: Tumor necrosis factor
FUS: Flora Ultra Strength
INU: Inulin
ROS: Rosavin
T.bilirubin: Total bilirubin
GGT: Gamma-glutamyl transferase
ALP: Alkaline phosphatase
TG: Triglycerides
TC: Total cholesterol
HDL-C: High-density lipoprotein cholesterol
LDL-C: Low-density lipoprotein cholesterol
D. bilirubin: Direct bilirubin
cDNA: Complementary DNA
DEGs: Differentially Expressed genes
GEO: Gene Expression Omnibus
PPI: Protein-protein interactions
HSHF: High sucrose and high-fat
H&E: Hematoxylin and eosin
MMPs: Matrix metalloproteinases
ECM: Extracellular matrix

## Footnotes

1 https://www.genome.jp/kegg/disease/

2 https://www.ncbi.nlm.nih.gov/geo/

3 http://microbiota.wall.gu.se/

4 http://mirwalk.umm.uni-heidelberg.de/

5 www.microrna.gr/LncBase

## Data availability

No datasets were generated or analysed during the current study.

## References

[1] Méndez-Sánchez N, Bugianesi E, Gish RG, Lammert F, Tilg H, Nguyen MH, et al. Global multi-stakeholder endorsement of the MAFLD definition. Lancet Gastroenterol Hepatol Elsevier. 2022;7:388–90. 10.1016/S2468-1253(22)00062-0 35248211

[2] Chan KE, Koh TJL, Tang ASP, Quek J, Yong JN, Tay P, et al. Global prevalence and clinical characteristics of metabolic-associated fatty liver disease: a meta-analysis and systematic review of 10 739 607 individuals. J Clin Endocrinol Metab. 2022;107:2691–700. Oxford University Press US. 35587339 10.1210/clinem/dgac321

[3] Eslam M, Newsome PN, Sarin SK, Anstee QM, Targher G, Romero-Gomez M, et al. A new definition for metabolic dysfunction-associated fatty liver disease: an international expert consensus statement. J Hepatol Elsevier. 2020;73:202–9. 10.1016/j.jhep.2020.03.039 32278004

[4] Bril F. What the new definition of Metabolic Dysfunction-Associated Fatty Liver Disease (MAFLD) left behind: Genetically Acquired Fatty Liver Disease (GAFLD). EBioMedicine. 2021;72:103584. 10.1016/j.ebiom.2021.103584. 10.1016/j.ebiom.2021.103584 PMC8479614 34563920

[5] Pirola CJ, Sookoian S. Metabolic dysfunction-associated fatty liver disease: advances in genetic and epigenetic implications. Curr Opin Lipidol. 2022;33:95–102. Wolters Kluwer. 34966133 10.1097/MOL.0000000000000814

[6] Xu R, Pan J, Zhou W, Ji G, Dang Y. Recent Advances in lean NAFLD. Biomed Pharmacother. 2022;153:113331. Elsevier. 35779422 10.1016/j.biopha.2022.113331

[7] Shi Y, Fan J. Therapeutic developments in metabolic dysfunction-associated fatty liver disease. Chin Med J (Engl). 2022;135:1009–18. Chinese Medical Journals Publishing House Co., Ltd. 42 Dongsi Xidajie. 35234696 10.1097/CM9.0000000000002091 PMC9276260

[8] Yip TC-F, Lyu F, Lin H, Li G, Yuen P-C, Wong VW-S, et al. Non-invasive biomarkers for liver inflammation in non-alcoholic fatty liver disease: present and future. Clin Mol Hepatol. 2023;29:S171. Korean Association for the Study of the Liver. 36503204 10.3350/cmh.2022.0426 PMC10029958

[9] Pierantonelli I, Svegliati-Baroni G. Nonalcoholic fatty liver disease: basic pathogenetic mechanisms in the progression from NAFLD to NASH. Transplantation. 2019;103:E1–13. 30300287 10.1097/TP.0000000000002480

[10] Chuah K-H, Chan W-K. Noninvasive biomarkers for liver inflammation in NAFLD: current and future. Clin Mol Hepatol. 2023;29:401–3. The Korean Association for the Study of the Liver. 36858457 10.3350/cmh.2023.0062 PMC10121314

[11] Chan W-K, Nik Mustapha NR, Mahadeva S. A novel 2-step approach combining the NAFLD fibrosis score and liver stiffness measurement for predicting advanced fibrosis. Hepatol Int Springer. 2015;9:594–602. 10.1007/s12072-014-9596-7 25788185

[12] Chan W, Treeprasertsuk S, Imajo K, Nakajima A, Seki Y, Kasama K, et al. Clinical features and treatment of nonalcoholic fatty liver disease across the Asia Pacific region—The GO ASIA initiative. Aliment Pharmacol Ther. 2018;47:816–25. Wiley Online Library. 29333610 10.1111/apt.14506

[13] Chalasani N, Younossi Z, Lavine JE, Charlton M, Cusi K, Rinella M, et al. The diagnosis and management of nonalcoholic fatty liver disease: practice guidance from the American association for the study of liver diseases. Hepatology. 2018;67(1):328–57. 10.1002/hep.29367. 28714183 10.1002/hep.29367

[14] Bril F, Ntim K, Lomonaco R, Cusi K. Treatment of nonalcoholic fatty liver disease (NAFLD) and nonalcoholic steatohepatitis (NASH). Int Textb Diabetes Mellit. 4th ed. Wiley Online Library; 2015. p. 292–305.

[15] Omokaro SO, Golden JK. The regulatory state of nonalcoholic steatohepatitis and metabolism. Endocrinol Diabetes Metab. 2020;3:e00113. Wiley-Blackwell. 33102795 10.1002/edm2.113 PMC7576228

[16] Loomba R, Ratziu V, Harrison SA, McFarlane SC, Tamaki N, Abdelmalek MF, et al. Expert panel review to compare FDA and EMA guidance on drug development and endpoints in nonalcoholic steatohepatitis. Gastroenterology. 2022;162:680–8. Elsevier. 34822801 10.1053/j.gastro.2021.10.051 PMC9683540

[17] Sanyal AJ, Friedman SL, McCullough AJ, Dimick-Santos L. Challenges and opportunities in drug and biomarker development for nonalcoholic steatohepatitis: Findings and recommendations from an American Association for the Study of Liver Diseases–US Food and Drug Administration Joint Workshop. Hepatology. 2015;61:1392–405. Wiley Online Library. 25557690 10.1002/hep.27678 PMC4900161

[18] Ratziu V, Francque S, Sanyal A. Breakthroughs in therapies for NASH and remaining challenges. J Hepatol. 2022;76:1263–78. Elsevier. 35589249 10.1016/j.jhep.2022.04.002

[19] Liu Q, Liu S, Chen L, Zhao Z, Du S, Dong Q, et al. Role and effective therapeutic target of gut microbiota in NAFLD/NASH. Exp Ther Med. 2019;18:1935–44. Spandidos Publications. 31410156 10.3892/etm.2019.7781 PMC6676192

[20] Guo J, Xu Y, Chen L, Zhang S, Liou Y, Chen X, et al. Gut microbiota and host Cyp450s co-contribute to pharmacokinetic variability in mice with non-alcoholic steatohepatitis: effects vary from drug to drug. J Adv Res. 2022;39:319–32. Elsevier. 35777915 10.1016/j.jare.2021.10.004 PMC9263650

[21] Li H, Xi Y, Xin X, Tian H, Hu Y. Salidroside improves high-fat diet-induced non-alcoholic steatohepatitis by regulating the gut microbiota–bile acid–farnesoid X receptor axis. Biomed Pharmacother. 2020;124:109915. Elsevier. 31986416 10.1016/j.biopha.2020.109915

[22] Wong VW-S, Wong GL-H, Chim AM-L, Chu WC-W, Yeung DK-W, Li KC-T, et al. Treatment of nonalcoholic steatohepatitis with probiotics. A proof-of-concept study. Ann Hepatol. 2013;12:256–62. Elsevier. 23396737 10.1016/S1665-2681(19)31364-X

[23] Ebrahimzadeh Leylabadlo H, Ghotaslou R, Samadi Kafil H, Feizabadi MM, Moaddab SY, Farajnia S, et al. Non-alcoholic fatty liver diseases: from role of gut microbiota to microbial-based therapies. Eur J Clin Microbiol Infect Dis. 2020;39:613–27. Springer. 31828683 10.1007/s10096-019-03746-1

[24] Vrieze A, Van Nood E, Holleman F, Salojärvi J, Kootte RS, Bartelsman JFWM, et al. Transfer of intestinal microbiota from lean donors increases insulin sensitivity in individuals with metabolic syndrome. Gastroenterology. 2012;143:913–6. Elsevier. 22728514 10.1053/j.gastro.2012.06.031

[25] Zhou D, Pan Q, Shen F, Cao H, Ding W, Chen Y, et al. Total fecal microbiota transplantation alleviates high-fat diet-induced steatohepatitis in mice via beneficial regulation of gut microbiota. Sci Rep. 2017;7:1529. Nature Publishing Group UK London. 28484247 10.1038/s41598-017-01751-y PMC5431549

[26] Harrison SA, Loomba R, Dubourg J, Ratziu V, Noureddin M. Clinical Trial Landscape in NASH. Clin Gastroenterol Hepatol. 2023;21:2001–14. Elsevier. 10.1016/j.cgh.2023.03.041 37059159

[27] Fraile JM, Palliyil S, Barelle C, Porter AJ, Kovaleva M. Non-Alcoholic Steatohepatitis (NASH) – a review of a crowded clinical landscape, driven by a complex disease. Drug Des Devel Ther. 2021;15:3997–4009. Taylor & Francis. 34588764 10.2147/DDDT.S315724 PMC8473845

[28] Mato JM, Alonso C, Noureddin M, Lu SC. Biomarkers and subtypes of deranged lipid metabolism in non-alcoholic fatty liver disease. World J Gastroenterol. 2019;25:3009. Baishideng Publishing Group Inc. 31293337 10.3748/wjg.v25.i24.3009 PMC6603806

[29] Chen Z, Yu Y, Cai J, Li H. Emerging molecular targets for treatment of nonalcoholic fatty liver disease. Trends Endocrinol Metab. 2019;30:903–14. Elsevier. 31597607 10.1016/j.tem.2019.08.006

[30] Musso G, Cassader M, Gambino R. Non-alcoholic steatohepatitis: emerging molecular targets and therapeutic strategies. Nat Rev Drug Discov. 2016;15:249–74. Nature Publishing Group UK London. 26794269 10.1038/nrd.2015.3

[31] Domínguez-Oliva A, Hernández-Ávalos I, Martínez-Burnes J, Olmos-Hernández A, Verduzco-Mendoza A, Mota-Rojas D. The importance of animal models in biomedical research: current insights and applications. Animals (Basel). 2023;13(7):1223. 10.3390/ani13071223. 37048478 10.3390/ani13071223 PMC10093480

[32] Hernandez LM, Blazer DG, editors. Washington (DC): Institute of Medicine (US) Committee on Assessing Interactions Among Social, Behavioral, and Genetic Factors in Health. Genes, Behavior, and the Social Environment: Moving Beyond the Nature/Nurture Debate. National Academies Press (US); 2006. PMID: 20669442. 20669442

[33] Salah N, Eissa S, Mansour A, El Magd NMA, Hasanin AH, El Mahdy MM, et al. Evaluation of the role of kefir in management of non-alcoholic steatohepatitis rat model via modulation of NASH linked mRNA-miRNA panel. Sci Rep. 2023;13:1–14. Nature Publishing Group UK. Available from: 10.1038/s41598-022-27353-x. 36604518 10.1038/s41598-022-27353-x PMC9816104

[34] Gadallah SH, Eissa S, Ghanem HM, Ahmed EK, Hasanin AH, El Mahdy MM, et al. Probiotic-prebiotic-synbiotic modulation of (YAP1, LATS1 and NF2 mRNAs/miR-1205/lncRNA SRD5A3-AS1) panel in NASH animal model. Biomed Pharmacother. 2021;140:111781. Available from: https://linkinghub.elsevier.com/retrieve/pii/S0753332221005631. 34090052 10.1016/j.biopha.2021.111781

[35] Hany NM, Eissa S, Basyouni M, Hasanin AH, Aboul-Ela YM, Elmagd NMA, et al. Modulation of hepatic stellate cells by Mutaflor® probiotic in non-alcoholic fatty liver disease management. J Transl Med. 2022;20(1):342. 35907883 10.1186/s12967-022-03543-z PMC9338485

[36] Albadawy R, Hasanin AH, Agwa SHA, Hamady S, Aboul-Ela YM, Raafat MH, et al. Rosavin ameliorates hepatic inflammation and fibrosis in the NASH rat model via targeting hepatic cell death. Int J Mol Sci. 2022;23:10148. Available from: https://www.mdpi.com/1422-0067/23/17/10148. 36077546 10.3390/ijms231710148 PMC9456245

[37] Huang AA, Huang SY. Increasing transparency in machine learning through bootstrap simulation and shapely additive explanations. PLoS One. 2023;18(2):e0281922. 10.1371/journal.pone.0281922. 10.1371/journal.pone.0281922 PMC9949629 36821544

[38] Christoph M. Interpretable machine learning a guide for making black box models explainable. Book. 2020.

[39] Libbrecht MW, Noble WS. Machine learning applications in genetics and genomics. Nat Rev Genet. 2015;16:321–32. Nature Publishing Group UK London. 25948244 10.1038/nrg3920 PMC5204302

[40] Zuo Y, Huang G, Nie S. Application and challenges of deep learning in the intelligent processing of medical images. J Image Graph. 2021.

[41] Lundberg SM, Lee SI. A unified approach to interpreting model predictions. Adv Neural Inf Process Syst. 2017;30.

[42] Huang AA, Huang SY. Use of machine learning to identify risk factors for insomnia. PLoS One. 2023;18(4):e0282622. 10.1371/journal.pone.0282622. 10.1371/journal.pone.0282622 PMC10096447 37043435

[43] Firoozbakht F, Yousefi B, Schwikowski B. An overview of machine learning methods for monotherapy drug response prediction. Brief Bioinform. 2022;23:bbab408. Oxford University Press. 34619752 10.1093/bib/bbab408 PMC8769705

[44] Sala E. Abstract IA04: Clinical challenges in oncologic imaging: AI support from image analysis to integrated diagnostics. Clin Cancer Res. 2020;26(12_Supplement_1):IA04.

[45] Turki T, Wang JTL. Clinical intelligence: new machine learning techniques for predicting clinical drug response. Comput Biol Med. 2019;107:302–22. Elsevier. 30771879 10.1016/j.compbiomed.2018.12.017

[46] Guo JH, Han DW, Li XQ, Zhang Y, Zhao YC. The impact of small doses of LPS on NASH in high sucrose and high fat diet induced rats. Eur Rev Med Pharmacol Sci. 2014;18:2742–7. 25317812

[47] Alves CC, Waitzberg DL, de Andrade LS, dos Santos Aguiar L, Reis MB, Guanabara CC, et al. Prebiotic and Synbiotic Modifications of Beta Oxidation and Lipogenic Gene Expression after Experimental Hypercholesterolemia in Rat Liver. Front Microbiol. 2017;8. Available from: 10.3389/fmicb.2017.02010/full. PMC5650986 29089934

[48] Abhari K, Shekarforoush SS, Sajedianfard J, Hosseinzadeh S, Nazifi S. The effects of probiotic, prebiotic and synbiotic diets containing Bacillus coagulans and inulin on rat intestinal microbiota. Iran J Vet Res. 2015;16:267–73. 27175187 PMC4782696

[49] Kosakova D, Scheer P, Lata J, Doubek J. Influence of the Escherichia coli Nissle 1917 strain on complications of chronic experimental liver damage. Vet Med (Praha). 2007;52:121–9. 10.17221/2013-VETMED

[50] Punaro GR, Maciel FR, Rodrigues AM, Rogero MM, Bogsan CSB, Oliveira MN, et al. Kefir administration reduced progression of renal injury in STZ-diabetic rats by lowering oxidative stress. Nitric Oxide. 2014;37:53–60. Available from: https://linkinghub.elsevier.com/retrieve/pii/S1089860313003571. 24406684 10.1016/j.niox.2013.12.012

[51] Li D, Park S, Lee K, Jang DS, Kim SK. 5-HT1A receptors mediate the analgesic effect of rosavin in a mouse model of oxaliplatin-induced peripheral neuropathic pain. Korean J Physiol Pharmacol. 2021;25(5):489–94. 10.4196/kjpp.2021.25.5.489. 34448466 10.4196/kjpp.2021.25.5.489 PMC8405433

[52] Juluri R, Vuppalanchi R, Olson J, Ünalp A, Van Natta ML, Cummings OW, et al. Generalizability of the NASH CRN histological scoring system for nonalcoholic fatty liver disease. J Clin Gastroenterol. 2011;45:55–8. NIH Public Access. 20505526 10.1097/MCG.0b013e3181dd1348 PMC2978283

[53] Chen B, Ma Y, Xue X, Wei J, Hu G, Lin Y. Tetramethylpyrazine reduces inflammation in the livers of mice fed a high fat diet. Mol Med Rep. 2019;19(4):2561–8. 10.3892/mmr.2019.9928. 30720104 10.3892/mmr.2019.9928 PMC6423567

[54] Livak KJ, Schmittgen TD. Analysis of relative gene expression data using real-time quantitative PCR and the 2-ΔΔCT method. Methods. 2001;25:402–8. 11846609 10.1006/meth.2001.1262

[55] Theobald O. Machine learning for absolute beginners: a plain english introduction. UK: Scatterplot Press London; 2017.

[56] Phyu TZ, Oo NN. Performance comparison of feature selection methods. MATEC Web Conf. 2016;42:6002. EDP Sciences.

[57] Kumar S, Chong I. Correlation analysis to identify the effective data in machine learning: prediction of depressive disorder and emotion states. Int J Environ Res Public Health. 2018;15:2907. Available from: http://www.mdpi.com/1660-4601/15/12/2907. 30572595 10.3390/ijerph15122907 PMC6313491

[58] Huang AA, Huang SY. Computation of the distribution of model accuracy statistics in machine learning: comparison between analytically derived distributions and simulation-based methods. Heal Sci Rep. 2023;6(4):e1214. 10.1002/hsr2.1214. 10.1002/hsr2.1214 PMC10119581 37091362

[59] Ho TK. Random decision forests. Proc 3rd Int Conf Doc Anal Recognit, vol. 1. Montreal: IEEE; 1995. p. 278–82.

[60] Breiman L. Bagging predictors. Mach Learn. 1996;24:123–40. Springer. 10.1007/BF00058655

[61] Breiman L. Statistical modeling: the two cultures (with comments and a rejoinder by the author). Stat Sci. 2001;16:199–231. Instituteof Mathematical Statistics. 10.1214/ss/1009213726

[62] Iverson LR, Prasad AM, Matthews SN, Peters M. Estimating potential habitat for 134 eastern US tree species under six climate scenarios. For Ecol Manage. 2008;254:390–406. Elsevier. 10.1016/j.foreco.2007.07.023

[63] Radhika PR, Nair RA, Veena G. A comparative study of lung cancer detection using machine learning algorithms. In: 2019 IEEE Int Conf Electr Comput Commun Technol. Coimbatore, India: IEEE; 2019. p. 1–4.

[64] Al-Zebari A, Sengur A. Performance comparison of machine learning techniques on diabetes disease detection. In: 2019 1st Int Informatics Softw Eng Conf. Ankara, Turkey: IEEE; 2019. p. 1–4.

[65] Li L, Wu Y, Ye M. Experimental comparisons of multi-class classifiers. Informatica. 2015;39:71–85.

[66] Azuaje F, Witten IH, Frank E. Data mining: practical machine learning tools and techniques 2nd edition. BBioMed Eng OnLine. 2006;5:51. 10.1186/1475-925X-5-51. 10.1186/1475-925X-5-51

[67] Murphy KP. Machine learning: a probabilistic perspective. MIT Press; 2012. Illustrated edition (24 August 2012).

[68] Burges CJC. A tutorial on support vector machines for pattern recognition. Data Min Knowl Discov. 1998;2:121–67. Springer. 10.1023/A:1009715923555

[69] Cortes C, Vapnik V. Support-Vector Networks. Mach Learn. 1995;20:273–97. Springer. 10.1007/BF00994018

[70] Aas K, Jullum M, Løland A. Explaining individual predictions when features are dependent: more accurate approximations to Shapley values. Artif Intell. 2021;298(2021):103502.

[71] Taylor-Weiner A, Pokkalla H, Han L, Jia C, Huss R, Chung C, et al. A machine learning approach enables quantitative measurement of liver histology and disease monitoring in NASH. Hepatology. 2021;74:133–47. Wiley Online Library. 33570776 10.1002/hep.31750 PMC8361999

[72] Yu SJ, Kim W, Kim D, Yoon J-H, Lee K, Kim JH, et al. Visceral obesity predicts significant fibrosis in patients with nonalcoholic fatty liver disease. Medicine (Baltimore). 2015;94:e2159. Wolters Kluwer Health. 26632897 10.1097/MD.0000000000002159 PMC4674200

[73] Nseir W, Hellou E, Assy N. Role of diet and lifestyle changes in nonalcoholic fatty liver disease. World J Gastroenterol WJG. 2014;20:9338–44. Baishideng Publishing Group Inc. 25071328 10.3748/wjg.v20.i28.9338 PMC4110565

[74] Heshmati HM. Gut microbiome and intestinal permeability are promising targets for treating nonalcoholic fatty liver disease. J Endocr Soc. 2021;5:A13–4. The Endocrine Society. 10.1210/jendso/bvab048.024

[75] Wagnerberger S, Spruss A, Kanuri G, Stahl C, Schröder M, Vetter W, et al. Lactobacillus casei Shirota protects from fructose-induced liver steatosis: a mouse model. J Nutr Biochem. 2013;24:531–8. Elsevier. 22749137 10.1016/j.jnutbio.2012.01.014

[76] Raso GM, Simeoli R, Iacono A, Santoro A, Amero P, Paciello O, et al. Effects of a Lactobacillus paracasei B21060 based synbiotic on steatosis, insulin signaling and toll-like receptor expression in rats fed a high-fat diet. J Nutr Biochem. 2014;25:81–90. Elsevier. 24314869 10.1016/j.jnutbio.2013.09.006

[77] Feng G, Zheng KI, Li Y, Rios RS, Zhu P, Pan X, et al. Machine learning algorithm outperforms fibrosis markers in predicting significant fibrosis in biopsy-confirmed NAFLD. J Hepato-Biliary-Pancreatic Sci 2021;28:593–603. Wiley Online Library. 10.1002/jhbp.972 33908180

[78] Chang D, Truong E, Noureddin M. Machine learning models for NAFLD/NASH and cirrhosis diagnosis and staging: accuracy and routine variables are the success keys. Hepatology. 2023;77(5):E105–6. 37018138 10.1097/HEP.0000000000000211

[79] Auslander N, Gussow AB, Koonin EV. Incorporating machine learning into established bioinformatics frameworks. Int J Mol Sci. 2021;22:2903. Available from: https://www.mdpi.com/1422-0067/22/6/2903. 33809353 10.3390/ijms22062903 PMC8000113

[80] Sorino P, Campanella A, Bonfiglio C, Mirizzi A, Franco I, Bianco A, et al. Development and validation of a neural network for NAFLD diagnosis. Sci Rep. 2021;11:20240. Nature Publishing Group UK London. 34642390 10.1038/s41598-021-99400-y PMC8511336

[81] Matboli M, Gadallah SH, Rashed WM, Hasanin AH, Essawy N, Ghanem HM, et al. mRNA-miRNA-lncRNA regulatory network in nonalcoholic fatty liver disease. Int J Mol Sci. 2021;22(13):6770. 10.3390/ijms22136770. 34202571 10.3390/ijms22136770 PMC8269036

[82] Nair B, Nath LR. Inevitable role of TGF-β1 in progression of nonalcoholic fatty liver disease. J Recept Signal Transduct. 2020;40:195–200. Available from: https://www.tandfonline.com/doi/full/10.1080/10799893.2020.1726952. 10.1080/10799893.2020.1726952 32054379

[83] Lu S, Wang Y, Liu J. Tumor necrosis factor-α signaling in nonalcoholic steatohepatitis and targeted therapies. J Genet Genomics. 2022;49:269–78. Available from: https://linkinghub.elsevier.com/retrieve/pii/S1673852721003374. 34757037 10.1016/j.jgg.2021.09.009

[84] Xu RY, Wan YP, Fang QY, Lu W, Cai W. Supplementation with probiotics modifies gut flora and attenuates liver fat accumulation in rat nonalcoholic fatty liver disease model. J Clin Biochem Nutr. 2012;50:72–7. 22247604 10.3164/jcbn.11-38 PMC3246186

[85] Alptekin İM, Çakıroğlu FP, Kiremitci S, Reçber T, Nemutlu E. Inulin may prevent steatosis by suppressing cannabinoid receptor-1 and patatin-like phospholipase-3 expression in liver. Nutrition. 2022;103–104:111742. 10.1016/j.nut.2022.111742. 10.1016/j.nut.2022.111742 35908495

[86] Yin Y, Liu H, Zheng Z, Lu R, Jiang Z. Genistein can ameliorate hepatic inflammatory reaction in nonalcoholic steatohepatitis rats. Biomed Pharmacother. 2019;111:1290–6. 10.1016/j.biopha.2019.01.004. 30841442 10.1016/j.biopha.2019.01.004

[87] Tsuchiya T, Naitoh T, Nagao M, Tanaka N, Watanabe K, Imoto H, et al. Increased bile acid signals after Duodenal-Jejunal bypass improve non-alcoholic steatohepatitis (NASH) in a rodent model of diet-induced NASH. Obes Surg. 2018;28(6):1643–52. 10.1007/s11695-017-3065-z. 29235014 10.1007/s11695-017-3065-z

[88] Gerhard GS, DiStefano JK. Micro RNAs in the development of non-alcoholic fatty liver disease. World J Hepatol. 2015;7(2):226. 25729477 10.4254/wjh.v7.i2.226 PMC4342604

[89] Sulaiman SA, Muhsin NIA, Jamal R. Regulatory non-coding RNAs network in non-alcoholic fatty liver disease. Front Physiol. 2019;10:1–11. 30941061 10.3389/fphys.2019.00279 PMC6433939

[90] Baffy G. MicroRNAs in nonalcoholic fatty liver disease. J Clin Med. 2015;4:1977–88. 26690233 10.3390/jcm4121953 PMC4693153

[91] Chen Y, Huang H, Xu C, Yu C, Li Y. Long non-coding RNA profiling in a non-alcoholic fatty liver disease rodent model: new insight into pathogenesis. Int J Mol Sci. 2017;18:21. Available from: http://www.mdpi.com/1422-0067/18/1/21. 28275212 10.3390/ijms18010021 PMC5297656

[92] Huang AA, Huang SY. Increasing transparency in machine learning through bootstrap simulation and shapely additive explanations. PLoS One. 2023;18:1–15. Available from: 10.1371/journal.pone.0281922 . 10.1371/journal.pone.0281922 PMC9949629 36821544

[93] Schomaker S, Potter D, Warner R, Larkindale J, King N, Porter AC, et al. Serum glutamate dehydrogenase activity enables early detection of liver injury in subjects with underlying muscle impairments. PLoS One. 2020;15:e0229753. Public Library of Science San Francisco, CA USA. 32407333 10.1371/journal.pone.0229753 PMC7224523

[94] Ma H, Xu CF, Shen Z, Yu CH, Li YM. Application of machine learning techniques for clinical predictive modeling: a cross-sectional study on nonalcoholic fatty liver disease in China. Biomed Res Int. 2018;2018:4304376. 10.1155/2018/4304376. 10.1155/2018/4304376 PMC6192080 30402478

[95] Fialoke S, Malarstig A, Miller MR, Dumitriu A. Application of machine learning methods to predict non-alcoholic steatohepatitis (NASH) in non-alcoholic fatty liver (NAFL) patients. AMIA Annu Symp Proc. American Medical Informatics Association; 2018;2018:430–9. PMC6371264 30815083

[96] Costello JC, Heiser LM, Georgii E, Gönen M, Menden MP, Wang NJ, et al. A community effort to assess and improve drug sensitivity prediction algorithms. Nat Biotechnol. 2014;32:1202–12. Nature Publishing Group US New York. 24880487 10.1038/nbt.2877 PMC4547623

[97] Ali M, Aittokallio T. Machine learning and feature selection for drug response prediction in precision oncology applications. Biophys Rev. 2019;11:31–9. Springer. 30097794 10.1007/s12551-018-0446-z PMC6381361

[98] Stetson LC, Pearl T, Chen Y, Barnholtz-Sloan JS. Computational identification of multi-omic correlates of anticancer therapeutic response. BMC Genomics. 2014;15:1–8. BioMed Central. 25573145 10.1186/1471-2164-15-S7-S2 PMC4243102

