# Supplementary material for: Machine learning based identification potential feature genes for prediction of drug efficacy in nonalcoholic steatohepatitis animal model
Source: Lipids Health Dis. 2024 Aug 24;23:266. doi: 10.1186/s12944-024-02231-9 (PMC11344433; doi:10.1186/s12944-024-02231-9)
Supplement: Supplementary file 1 — Supplementary Material 1. Fig. A.1. Showing the pathogenic pathways and processes involved in NAFLD/NASH genesis through the KEGG pathway database, Fig. A.2. showing the involvement of the biochemical-RNA signatures in pathogenic mechanisms (Hippo signaling, TGF-β signaling, TNF signaling pathway, apoptosis, oxidative stress, and inflammatory response) through the KEGG pathway database, and GeneCards database; Fig. A.3. Validation that our selected mRNAs are key regulatory genes in gut microbiota, Fig. A.4. Validation of the interaction between the selected mRNAs and the retrieved miRNAs from mirwalk3; Fig. A.5. Validation of the relation of the candidate miRNAs to pathogenic mechanisms such as Hippo signaling, and TGF-β signaling through DIANA tools mirPath 3; Fig. A.6. Validation of the interaction between the selected miRNAs and the retrieved lncRNAs from mirwalk3 and DIANA-LncBase; Table A.1. The detailed differentially expressed genes in NASH were retrieved from the gene chip datasets GSE164760, GSE24807, and GSE126848, Table A.2. List of primer assays; Table A.3. Histopathological scoring grid for NAFLD/NASH liver sections. [file 12944_2024_2231_MOESM1_ESM.zip › Suppl table & figures.docx]

|  | **Normal** | **NASH-12wk** | **NASH-9wk** | **FUS** | **INU** | **FUS+INU** | **Mutaflor** | **Kefir** | **Ros-10** | **Ros-20** | **Ros-30** |
| --- | --- | --- | --- | --- | --- | --- | --- | --- | --- | --- | --- |
| **ALT** **(U/L)** | 34.6 ± 5.02 | 131.2 ± 16.62^*^ | 83.7 ± 9.48^*##^ | 63.7 ± 6.80^##a^ | 68.6 ± 7.77^##a^ | 48.3 ± 6.34^##a^ | 50.6 ± 7.01^##a^ | 41 ± 5.49^##a^ | 80.2 ± 7.05^##^ | 48.3 ± 11.61^##a^ | 22.5 ± 5.55^##a^ |
| **AST (U/L)** | 30.1 ± 6.96 | 91.6 ± 9.19^*^ | 76.8 ± 7.67^*##^ | 60 ± 4.43^##a^ | 63.6 ± 6.75^##a^ | 50.5 ± 7.56^##a^ | 43.1 ± 5.72^##a^ | 35.1 ± 4.85^##a^ | 87.3 ± 10.4^a^ | 57.9 ± 4.44^##a^ | 36.9 ± 4.25^##a^ |
| **ALP (U/L)** | 33.7 ± 6.07 | 123.8 ± 9.63^*^ | 100.5 ± 11.01^*##^ | 55.1 ± 7.58^##a^ | 64.3 ± 8.02^##a^ | 52.9 ± 7.13^##a^ | 65.4 ± 7.21^##a^ | 52.8 ± 6.20^##a^ | 86.5 ± 6.67^##a^ | 70.2 ± 5.51^##a^ | 51.8 ± 5.88^##a^ |
| **GGT (U/L)** | 16.0 ± 3.20 | 70.5 ± 13.67^*^ | 63.8 ± 12.91^*^ | 32.5 ± 3.90^##a^ | 33.9 ± 5.08^##a^ | 20.6 ± 4.56^##a^ | 39.6 ± 5.72^##a^ | 32.4 ± 4.80^##a^ | 49.5 ± 7.49^##a^ | 44.6 ± 11.09^##a^ | 33.1 ± 4.83^##a^ |
| **T.Bilirubin (mg/dL)** | 0.30 ± 0.069 | 1.61 ± 0.24^*^ | 1.18 ± 0.21^*##^ | 0.58 ± 0.09^##a^ | 0.65 ± 0.08^##a^ | 0.5 ± 0.11^##a^ | 0.56 ± 0.09^##a^ | 0.47 ± 0.07^##a^ | 2.7 ± 0.24^##a^ | 1.7 ± 0.13^a^ | 0.69 ± 0.16^##a^ |
| **D.Bilirubin (mg/dL)** | 0.15 ± 0.03 | 0.75 ± 0.15^*^ | 0.52 ± 0.12^*##^ | 0.27 ± 0.03^##a^ | 0.30 ± 0.03^##a^ | 0.23 ± 0.03^##a^ | 0.27 ± 0.06^##a^ | 0.22 ± 0.05^##a^ | 1.7 ± 0.38^##a^ | 1.08 ± 0.16^##a^ | 0.47 ± 0.11^##^ |
| **AFP** | 20.0 ± 3.48 | 535.7 ± 81.78^*^ | 475.1 ± 61.60^*#^ | 95.2 ± 16.78^##a^ | 109.9 ± 16.78^##a^ | 29 ± 6.65^##a^ | 29.6 ± 6.53^##a^ | 34.6 ± 5.3^##a^ | 211.3 ± 25.89^##a^ | 99.0 ± 18.32^##a^ | 29.5 ± 6.53^##a^ |
| **Albumin (g/dL)** | 1.8 ± 0.28 | 3.6 ± 0.313^*^ | 3.4 ± 0.46^*^ | 2.6 ± 0.174^##a^ | 2.9 ± 0.17^##a^ | 2.1 ± 0.37^##a^ | 2.0 ± 0.36^##a^ | 2.0 ± 0.34^##a^ | 2.9 ± 0.12^##a^ | 2.6 ± 0.18^##a^ | 1.9 ± 0.37^##a^ |
| **TC (mg/dL)** | 83.4 ± 11.77 | 152.3 ± 33.68^*^ | 130.7 ± 9.72^*#^ | 95.7 ± 16.90^##a^ | 92.7 ± 10.1^##a^ | 85.7 ± 4.99^##a^ | 110.1 ± 10.89^##a^ | 98.1 ± 9.74^##a^ | 193.4 ± 6.84^##a^ | 169.7 ± 7.19^a^ | 116 ± 19.78^##^ |
| **TG (mg/dL)** | 56.8 ± 13.17 | 188.4 ± 41.49^*^ | 116.8 ± 12.05^*##^ | 109.2 ± 8.68^##^ | 122.4 ± 20.8^##^ | 91.5 ± 10.67^##a^ | 81.2 ± 4.69^##a^ | 76.3 ± 5.84^##a^ | 99.4 ± 18.15^##^ | 77.2 ± 11.46^##a^ | 56.1 ± 4.72^##a^ |
| **HDL-C (mg/dL)** | 47.6 ± 11.10 | 27.2 ± 5.08^*^ | 34.9 ± 3.70^*#^ | 31.5 ± 3.26 | 30.7 ± 2.95 | 32.6 ± 3.20 | 55.2 ± 3.35^##a^ | 49 ± 4.05^##a^ | 33.7 ± 1.96^#^ | 39 ± 3.33^##^ | 45.2 ± 3.04^##a^ |
| **LDL-C (mg/dL)** | 16.6 ± 3.52 | 100.1 ± 19.58^*^ | 64.7 ± 8.64^*##^ | 36.3 ± 6.89^##a^ | 37.6 ± 7.13^##a^ | 33.5 ± 4.47^##a^ | 51.5 ± 7.25^##a^ | 47.8 ± 4.16^##a^ | 145.6 ± 8.07^##a^ | 115.8 ± 11.41^#a^ | 71.4 ± 9.12^##^ |
| **IL-6** | 1270 ± 230 | 4692 ± 392^*^ | 4033 ± 599^*##^ | 2412 ± 94^##a^ | 2631 ± 265^##a^ | 1948 ± 190^##a^ | 2234 ± 140^##a^ | 2000 ± 86^##a^ | 2660 ± 287^##a^ | 2415 ± 93^##a^ | 1869 ± 212^##a^ |
| **TGFB1** | 1740 ± 291 | 6512 ± 758^*^ | 4328 ± 437^*##^ | 3882 ± 170^##^ | 4188 ± 241^##^ | 3462 ± 225^##a^ | 2803 ± 433^##a^ | 2545 ± 400^##a^ | 4214 ± 184^##a^ | 3856 ± 220^##^ | 3454 ± 233^##^ |

Values are mean ± SD; the number of animals = 12 rats/each group except n=10 for the Normal group. *^*^ p < 0.001* compared to the Normal group. *^##^p < 0.001* and *^#^p < 0.05* compared to NASH-12wk group, *^a^ p < 0.05* compared to NASH-9wk. One-way ANOVA followed by Tukey’s multiple comparison test.

**Table S1.** The effect of Microbiome-targeted therapies and herbal rosavin on the serum biochemicals and the hepatic target effector signatures corresponding to HPS levels.

| **Before UpSampling (130 rows)** | | | | | | **After UpSampling (270 rows)** | | | |
| --- | --- | --- | --- | --- | --- | --- | --- | --- | --- |
| Molecular Features Only 20 Columns | Model | Accuracy | Test accuracy | Precision | Recall | Accuracy | Test accuracy | Precision | Recall |
|  | Logistic | 70.19 | 65.38 | 76.79 | 65.38 | 77.778 | 83.33 | 92.10 | 83.33 |
|  | KNN | 66.35 | 65.38 | 77.78 | 65.38 | 83.80 | 75.93 | 76.77 | 75.93 |
|  | DT | 89.42 | 65.38 | 80.91 | 65.38 | 94.91 | 88.89 | 91.95 | 88.89 |
|  | RF | 89.42 | 76.92 | 76.15 | 76.92 | 94.91 | 90.74 | 93.80 | 90.74 |
|  | GBoost | 89.42 | 65.38 | 78.98 | 65.38 | 94.91 | 94.44 | 95.94 | 83.33 |
|  | SVC-LR | 72.12 | 73.08 | 80.45 | 73.08 | 79.17 | 83.33 | 92.10 | 90.74 |
| Biochemical Features Only 14 Columns | Model | Accuracy | Test accuracy | Precision | Recall | Accuracy | Test accuracy | Precision | Recall |
|  | Logistic | 75.00 | 46.15 | 56.35 | 46.15 | 87.96 | 79.63 | 82.15 | 79.63 |
|  | KNN | 69.23 | 53.85 | 51.24 | 53.85 | 82.87 | 85.19 | 89.28 | 85.19 |
|  | DT | 94.23 | 57.69 | 61.86 | 57.69 | 95.83 | 85.19 | 86.71 | 85.19 |
|  | RF | 94.23 | 53.85 | 56.04 | 53.85 | 95.83 | 83.33 | 77.70 | 83.33 |
|  | GBoost | 94.23 | 61.54 | 73.30 | 61.54 | 95.83 | 82.33 | 82.90 | 83.33 |
|  | SVC-LR | 81.73 | 57.69 | 65.77 | 57.69 | 89.35 | 90.74 | 91.10 | 90.74 |
| Molecular and biochemical combined 34 columns | Model | Accuracy | Test accuracy | Precision | Recall | Accuracy | Test accuracy | Precision | Recall |
|  | Logistic | 89.42 | 69.23 | 76.60 | 69.23 | 93.52 | 92.59 | 95.18 | 92.59 |
|  | KNN | 70.19 | 50.00 | 68.10 | 50.00 | 86.57 | 94.44 | 95.88 | 94.44 |
|  | DT | 98.08 | 57.69 | 66.39 | 57.69 | 99.07 | 96.30 | 97.12 | 96.30 |
|  | RF | 98.08 | 61.54 | 68.54 | 61.54 | 99.07 | 94.44 | 96.83 | 94.44 |
|  | GBoost | 98.08 | 57.69 | 61.86 | 57.69 | 99.07 | 98.15 | 98.38 | 98.15 |
|  | SVC-LR | 89.42 | 65.38 | 74.68 | 65.38 | 94.44 | 94.44 | 96.83 | 94.44 |

**Table S2.** Comparison of the performance of the different machine-learning algorithms

| Treatment | Study | ID | Period |
| --- | --- | --- | --- |
| Inulin | Modification of Gut Microbiota in the Treatment of Insulin Resistance: a Personalized Approach (TRIEMA) | NCT03710850 | Inulin was supplemented in a dose of 10g/d for a period of 3 months. |
| Kefir | Kefir and Metabolic Syndrome | NCT03966846 | Participant received one bottle of kefir (180 ml) daily for 12 weeks. |
| Rosavin | Rhodiola Rosea Therapy of Major Depressive Disorder | NCT01098318 | Herbal extract (340-1,360 mg/daily) was supplemented for 12 weeks |
| Probiotics | Probiotic Intervention for Microbiome Modifications and Clinical Improvements in Fragile X Syndrome | NCT06279858 | Each participant receives probiotic for 3 months (12 weeks) |

**Table S3:** Clinical trials on the applied treatment obtained from ClinicalTrials.gov: <https://clinicaltrials.gov/>


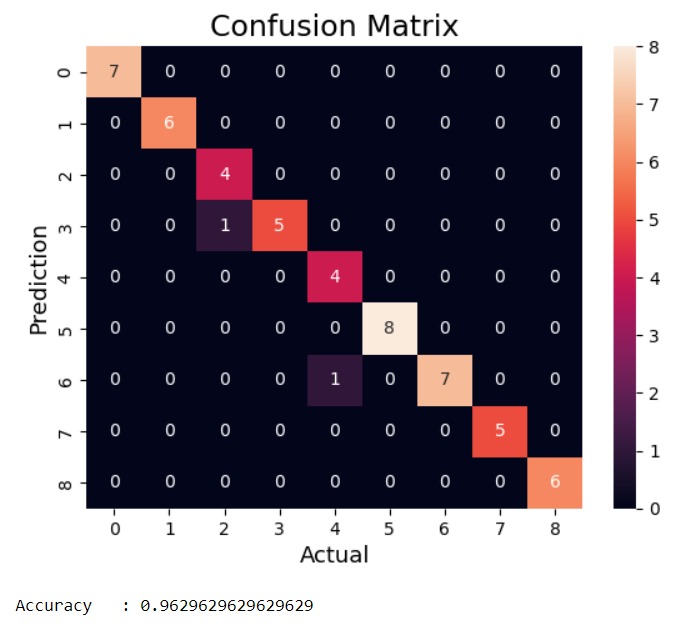


**Fig. S1** Confusion Matrix of GBoost Predictions


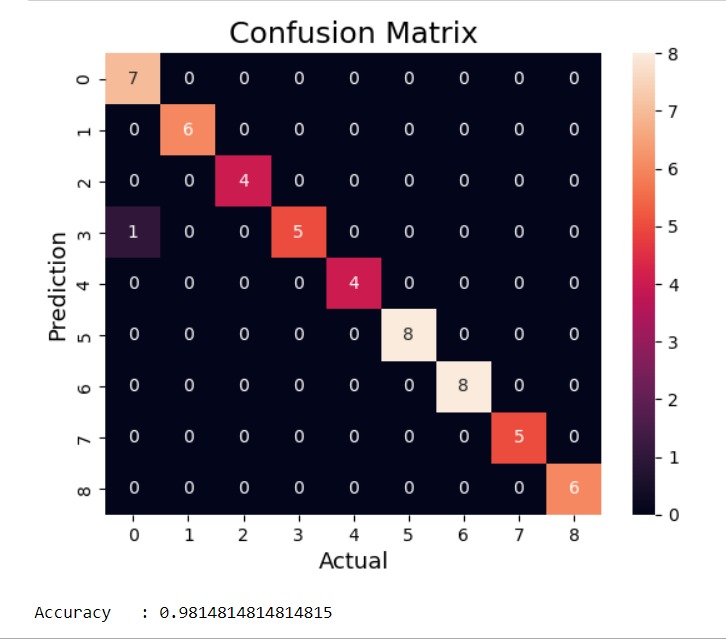


**Fig. S2** Confusion Matrix of Random Forest prediction on top features
